# Bicaudal D2, Dynein, and Kinesin-1 Associate with Nuclear Pore Complexes and Regulate Centrosome and Nuclear Positioning during Mitotic Entry

**DOI:** 10.1371/journal.pbio.1000350

**Published:** 2010-04-06

**Authors:** Daniël Splinter, Marvin E. Tanenbaum, Arne Lindqvist, Dick Jaarsma, Annette Flotho, Ka Lou Yu, Ilya Grigoriev, Dieuwke Engelsma, Elize D. Haasdijk, Nanda Keijzer, Jeroen Demmers, Maarten Fornerod, Frauke Melchior, Casper C. Hoogenraad, René H. Medema, Anna Akhmanova

**Affiliations:** 1 Department of Cell Biology, Erasmus Medical Center, Rotterdam, The Netherlands; 2 Department of Medical Oncology and Cancer Genomics Center, University Medical Center, Utrecht, The Netherlands; 3 Department of Neuroscience, Erasmus Medical Center, Rotterdam, The Netherlands; 4 Center for Molecular Biology Heidelberg (ZMBH), Heidelberg, Germany; 5 Division of Gene Regulation, The Netherlands Cancer Institute, Amsterdam, The Netherlands; 6 Proteomics Center, Erasmus Medical Center, Rotterdam, The Netherlands; Dana-Farber Cancer Institute, United States of America

## Abstract

BICD2 is one of the two mammalian homologues of the *Drosophila* Bicaudal D, an evolutionarily conserved adaptor between microtubule motors and their cargo that was previously shown to link vesicles and mRNP complexes to the dynein motor. Here, we identified a G2-specific role for BICD2 in the relative positioning of the nucleus and centrosomes in dividing cells. By combining mass spectrometry, biochemical and cell biological approaches, we show that the nuclear pore complex (NPC) component RanBP2 directly binds to BICD2 and recruits it to NPCs specifically in G2 phase of the cell cycle. BICD2, in turn, recruits dynein-dynactin to NPCs and as such is needed to keep centrosomes closely tethered to the nucleus prior to mitotic entry. When dynein function is suppressed by RNA interference-mediated depletion or antibody microinjection, centrosomes and nuclei are actively pushed apart in late G2 and we show that this is due to the action of kinesin-1. Surprisingly, depletion of BICD2 inhibits both dynein and kinesin-1-dependent movements of the nucleus and cytoplasmic NPCs, demonstrating that BICD2 is needed not only for the dynein function at the nuclear pores but also for the antagonistic activity of kinesin-1. Our study demonstrates that the nucleus is subject to opposing activities of dynein and kinesin-1 motors and that BICD2 contributes to nuclear and centrosomal positioning prior to mitotic entry through regulation of both dynein and kinesin-1.

## Abstract 2

Mammalian Bicaudal D2 is the missing molecular link between cytoplasmic motor proteins and the nucleus during nuclear positioning prior to the onset of mitosis.

## Author Summary

Bidirectional microtubule-based transport is responsible for the positioning of a large variety of cellular organelles, but the molecular mechanisms underlying the recruitment of microtubule-based motors to their cargoes and their activation remain poorly understood. In particular, the molecular players involved in the important processes of nuclear and centrosomal positioning prior to the onset of cell division are not known. In this study we focus on the function of one of the mammalian homologues of *Drosophila* Bicaudal D, an adaptor for the microtubule minus-end-directed dynein-dynactin motor complex. Previously, *Drosophila* Bicaudal D and its mammalian homologues were shown to act as linkers between the dynein motor and mRNP complexes or secretory vesicles. Here, we identify a new cargo for mammalian Bicaudal D2 (BICD2)–the nucleus. We show that BICD2 specifically binds to nuclear pore complexes in cells in G2 phase of the cell division cycle. We also show that this interaction is required for G2-specific recruitment of dynein to the nuclear envelope and thus for proper positioning of the nucleus relative to centrosomes prior to the onset of mitosis. Further, our findings demonstrate that the motor protein kinesin-1 opposes dynein's activity during this process and requires BICD2 for its activity. Our study therefore reveals BICD2 as the critical molecular adaptor that allows molecular motors to regulate nuclear and centrosomal positioning before cell division.

## Introduction

Spatial organization of eukaryotic cells requires active transport of proteins, macromolecular assemblies, and membrane organelles along cytoskeletal fibers. Transport is driven by motor proteins, which use actin and microtubules (MTs) as tracks for their movement. Cytoskeletal elements are polarized structures, and each particular motor can move along them only in one direction. For example, MT-based motors include kinesins, which with a few exceptions walk to MT plus ends, and dyneins, which drive minus end-directed transport [1].

Motor-dependent transport machineries display a high degree of complexity. First, the same motor can move multiple cargos. For example, cytoplasmic dynein is responsible for the movement of the majority of membrane organelles, mRNAs, and proteins to MT minus ends [1],[2]. Second, the same cargo can simultaneously associate with multiple motors of opposite polarity and frequently switch the direction of movement [3],[4]. Molecular mechanisms responsible for motor recruitment, activation, and switching of directions are still poorly understood. Motors are likely to be controlled by cargo-specific adaptor complexes, which often include structural components and small GTPases [5],[6].

An example of a well-studied motor adaptor is Bicaudal D (BICD), which is conserved throughout the animal kingdom [7]. BICD consists of several coiled coil segments separated by regions expected to be highly flexible. The N-terminal part of BICD binds to cytoplasmic dynein and its accessory factor dynactin; moreover, the BICD N-terminus is sufficient to recruit these complexes to organelles [8],[9]. The C-terminal domain of BICD is the cargo-binding part of the molecule. In mammals and flies, it directly associates with the small GTPase Rab6 [10]–[12]. In mammalian cells, BICD participates in recruitment of dynein/dynactin to Rab6-positive exocytotic vesicles and promotes their MT minus end-directed transport [11],[13]. The middle portion of BICD weakly binds to kinesin-1 [13]. The functional role of this link is not yet clear, but it is noteworthy that BICD-bound Rab6 vesicles move mostly towards the MT plus ends, suggesting that kinesin motor activity on Rab6 vesicles predominates over dynein-dependent transport [11],[13]. In *Drosophila*, BicD participates in dynein-dependent mRNP transport [14],[15]. This function depends on the association of BicD C terminus with the RNA-binding protein Egalitarian [15]–[17]. BicD is also involved in both dynein and kinesin-1-dependent movement of lipid droplets in fly embryos [18].

To investigate whether mammalian BICD is involved in other transport routes in addition to Rab6 vesicle trafficking, we searched for partners of the cargo-binding domain of BICD2, one of the two mammalian homologues of the fly BicD [8]. We identified a component of the nuclear pore complex (NPC), RanBP2 [19],[20], as the major interacting partner of BICD2 C terminus. RanBP2 (also known as NUP358) is a large protein, which acts as docking factor in nucleocytoplasmic transport [21] and is an E3 ligase for posttranslational modification with the ubiquitin-like protein SUMO1 [22]. RanBP2 exists in a tight complex with the sumoylated form of RanGAP1, the Ran GTPase-activation protein, and targets it to the NPCs [23],[24]. RanBP2 forms extended fibers at the cytoplasmic side of the NPC and represents a good candidate for a link between the cytoskeleton and the nuclear envelope (NE).

Previous studies showed that cytoplasmic dynein is specifically recruited to the NE in late G2/mitotic prophase, where it participates in NE breakdown (NEB) during mitotic entry ([25],[26]; for review see [27],[28]). Furthermore, NE-bound dynein is thought to pull centrosomes towards the NE, through its minus-end-directed motility, thereby contributing to proper attachment of centrosomes to the NE [29],[30]. In yeast, a dynein light chain is a nucleoporin, but it likely acts at the NPC independently of the dynein motor [31]. In *C. elegans*, dynein is anchored to the NE by the nuclear membrane component SUN-1 and a hook protein ZYG-12 [32]. Also in mammals, SUN1/2 and Syne/Nesprin-1/2 complexes, together with associated MT motors, are important to maintain the connection between the centrosome and nucleus during neuronal migration [33]. However, the molecular mechanism of G2-specific dynein interaction with the NE in dividing mammalian cells has not yet been clarified.

Here, we show that BICD2 specifically associates with the NPCs through RanBP2 in the G2 phase of the cell cycle and participates in the recruitment of the dynein/dynactin complexes to these structures. In addition, BICD2 associates with kinesin-1 [13] and we show that while dynein pulls centrosomes and the nucleus together during mitotic entry, kinesin-1 pushes them apart. During late G2, cytoplasmic dynein activity predominates over kinesin-1 activity, and the centrosomes remain tightly associated with the NE. Furthermore, we show that BICD2 not only acts to recruit dynein to the NE but is also required for the oppositely directed kinesin-1 activity, explaining why loss of BICD2 results only in a mild defect in centrosome-nuclear attachment. These results suggest that similar to most other MT motor cargos in animal cells, the prophase cell nucleus is transported bi-directionally by a molecular complex combining MT motors of opposite polarity.

## Results

### RanBP2 Directly Binds to BICD2 C Terminus

**Figure 1:**
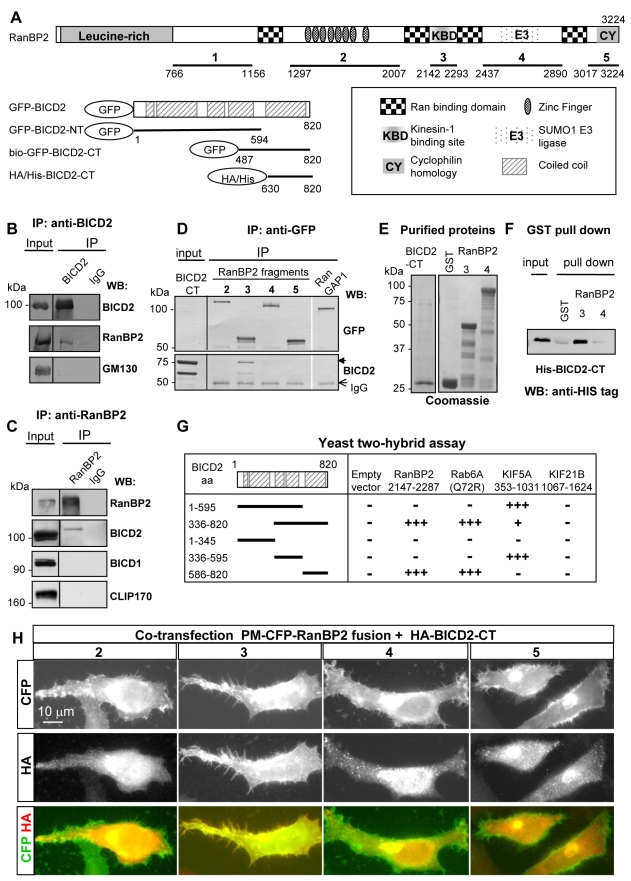
BICD2 interacts directly with RanBP2. (A) Schematic representation of the domains of RanBP2 and BICD2 and their fragments used for binding studies. (B) Co-IP of endogenous BICD2 and RanBP2 from HeLa cells. IPs were performed with antibodies against BICD2 or the control IgG and analyzed by Western blotting with the indicated antibodies. 2.5% of the input was loaded on gel. RanBP2, but not the negative control, GM130, is co-precipitated with BICD2. (C) Co-IP of endogenous RanBP2 and BICD2 from nocodazole-arrested HeLa cells. IPs were performed with antibodies against RanBP2 or the control IgG and analyzed by Western blotting with the indicated antibodies. 3% of the input was loaded on gel. BICD2 is co-precipitated with RanBP2, while its close homologue BICD1 and another dynactin-binding protein, CLIP-170, are not. (D) Co-IPs from HEK293 cells co-transfected with CFP-tagged RanBP2 fragments or GFP-RanGAP1 and mCherry-fused BICD2-CT. IPs were performed using mouse anti-GFP antibodies and analyzed by Western blotting using rabbit anti-GFP or anti-BICD2 antibodies. BICD2-CT and the IgG bands are indicated by an arrowhead and an arrow. BICD2-CT is coprecipitated with RanBP2 fragment 3, but not with other RanBP2 fragments or RanGAP1. (E) HIS-tagged BICD2-CT, GST, and GST fusions of RanBP2 fragments 3 and 4 were purified from *E. coli* and analyzed by SDS-polyacrilamide gel electrophoresis and Coomassie staining. (F) GST pull-down assays with the indicated RanBP2 fusions and purified HIS-tagged BICD2-CT, which was detected by Western blotting with anti-HIS tag antibodies. 10% of the input and the bound fractions were loaded on gel. Using 0.8 µM BICD2-CT and 1.4 µM GST-RanBP2 fragment 3, an almost quantitative binding was observed. This experiment confirms a direct interaction between RanBP2 fragment 3 and BICD2-CT. (G) Yeast two-hybrid analysis. BICD2 fragments were linked to GAL4 activation domain and tested in a pairwise fashion for interaction with RanBP2, GTP-bound Rab6A (Q72R), kinesin-1 (KIF5A), and kinesin-4 (KIF21B) tail regions cloned into LexA fusion vector. Interaction strength was scored according to the time needed for β-galactosidase reporter to generate visible blue-colored yeast colonies on X-Gal containing filters: +++0–30 min, ++30–60 min, +60–180 min and - no β-galactosidase activity. Similar to the previously characterized BICD2 partner Rab6, RanBP2 fragment 3 (2147–2287) specifically interacts with BICD2-CT, but not with other parts of BICD2. (H) HeLa cells were co-transfected with the indicated plasma membrane targeted CFP-RanBP2 fusions and HA-tagged BICD2-CT, fixed with paraformaldehyde, and stained with anti-HA antibodies. CFP fluorescence was visualized directly. CFP-tagged palmitoylated RanBP2 fragments are present on the plasma membrane (upper panels), while BICD2-CT (upper panels) localizes to the Golgi and cytoplasmic vesicles (middle panels). RanBP2 fragment 3, but not the other RanBP2 fragments, recruits BICD2-CT to the plasma membrane (see overlay in the bottom panels).

Our previous studies showed that the individual coiled coil segments of BICD2 display strong association with their binding partners, while the full-length molecule binds to the same proteins less efficiently, suggesting that it may be autoinhibited [8],[9],[11]. Therefore, we used the C-terminal coiled coil segment of BICD2 (Figure 1A) as a bait to search for new BICD2 cargos. We linked this BICD2 fragment to GFP and a biotinylation tag (Bio), a short peptide sequence that can be modified by the addition of biotin when expressed together with the biotin ligase BirA [34]. The resulting Bio-GFP-BICD2-CT fusion was transiently expressed together with BirA in HeLa cells, which were used for pull-down assays with streptavidin beads (Figure S1). The resulting protein complexes were analyzed by mass spectrometry (Table S1). The most abundant newly identified potential BICD2 partner was the NPC component RanBP2. RanGAP1, the sumoylated form of which is known to form a tight complex with RanBP2 [23],[24], was also present among the isolated proteins in highly significant amounts (Table S1).

The results of the pull-down assay were confirmed by co-immunoprecipitation (co-IP) of endogenous RanBP2 with endogenous BICD2; an abundant Golgi-associated protein GM130 served as a negative control (Figure 1B). Further, we observed co-IP of endogenous BICD2 with RanBP2 from nocodazole-arrested HeLa cells, but neither BICD1 nor CLIP-170, another cytosolic protein known to interact with dynactin [35], were coprecipitated with RanBP2 in these conditions (Figure 1C). Next, we investigated which domain of RanBP2 associated with BICD2. RanBP2 is a protein of ∼350 kDa, which contains a leucine-rich region, four Ran-binding domains, eight zinc finger motifs, and a C-terminal cyclophilin A-homologous region (Figure 1A). We generated expression constructs of five RanBP2 fragments, which covered most of the RanBP2 sequence, as fusions to CFP and the plasma membrane-targeting palmitoylation motif of GAP-43 (Figure 1A). With the exception of the N-terminal fragment 1, these fusions were expressed well in mammalian cells. Using co-IP from HEK293 cells, we found that the C-terminal domain of BICD2 specifically interacted with RanBP2 fragment 3 (Figure 1D). This experiment also showed that BICD2-CT does not interact with the overexpressed GFP-tagged RanGAP1 (Figure 1D), indicating that coprecipitation of RanBP2-RanGAP1 complex with BICD2-CT, observed by mass spectrometry, is due to BICD2 interaction with RanBP2. The interaction between BICD2 and RanBP2 is direct, since BICD2-CT and RanBP2 segment 3, purified from bacteria, specifically bind to each other in a glutathione S-transferase (GST) pull-down assay (Figure 1E,F). Remarkably, the same RanBP2 fragment was previously shown to interact directly with kinesin-1 isoforms KIF5B and KIF5C [36]–[38], supporting the notion that it is involved in MT motor recruitment.

Next, we employed a yeast two-hybrid assay, which showed that RanBP2 fragment 3 binds exclusively to the C-terminal part of BICD2 and not to its N-terminal and middle segments (Figure 1G). This is similar to the previously described interaction between BICD2 and Rab6 [8],[11] and is in contrast to kinesin-1 KIF5A, which associates with the middle portion of BICD2 (Figure 1G) [13].

Finally, we tested if fragment 3 of RanBP2 was sufficient to recruit BICD2 C terminus to ectopic sites within the cell. For this, RanBP2 fragments were artificially targeted to the plasma membrane through addition of a palmitoylation motif, as described above. The palmitoylation motif fusions of RanBP2 fragments displayed a strong association with the plasma membrane, including filopodia, and also with the Golgi complex (Figure 1H), but as expected, did not target to the NE. BICD2-CT expressed in mammalian cells associates with the Golgi and cytoplasmic vesicles, but not with the plasma membrane [8],[11]. Interestingly, BICD2-CT was specifically recruited to the plasma membrane by RanBP2 fragment 3 but not by other RanBP2 fragments (Figure 1H), suggesting that this domain of RanBP2 can serve as a recruitment factor for BICD2.

### RanBP2 Recruits BICD2 to NPCs during G2

**Figure 2:**
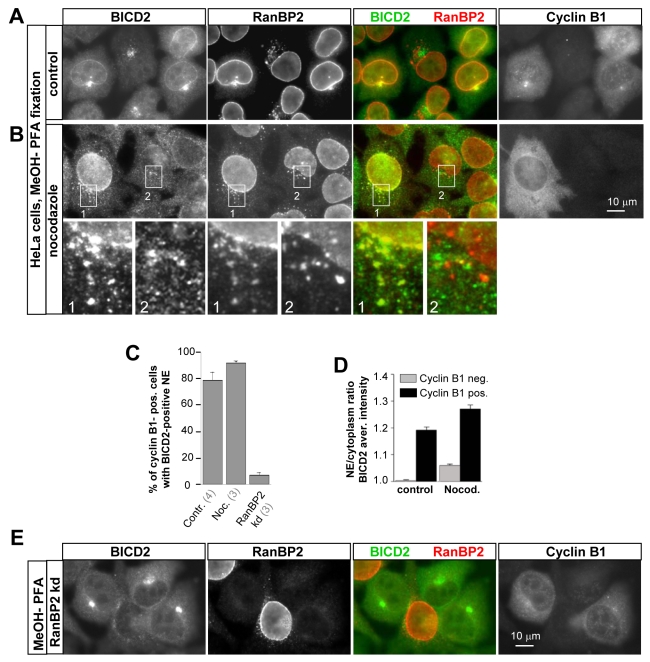
BICD2 associates with the NE and AL in G2 phase in a RanBP2-dependent manner. (A) HeLa cells were transfected with a control siRNA, fixed with cold methanol followed by paraformaldehyde 3 d later, and stained for endogenous BICD2, RanBP2, and cyclin B1. Note that in the cyclin B1-positive cell, BICD2 and RanBP2 co-localize at the NE and at a perinuclear spot that is an accumulation of AL. (B) HeLa cells were treated with 10 µM nocodazole for 1 h and stained as described for (A). Note that BICD2 strongly stains the NE and cytoplasmic NPC-positive dots (AL) in the cyclin B1-positive but not the cyclin B1 negative cells. (C) HeLa cells that were either treated with 10 µM nocodazole or transfected with control siRNAs or a mixture of RanBP2 siRNAs #1 and #2 were stained as described for (A), and the percentage of cyclin B1-positive cells showing BICD2 accumulation at the NE was counted. In case of RanBP2 knockdown, only the cells in which RanBP2-specific nuclear staining was reduced to background levels were included in the quantification. Error bars represent SD; the number of experiments for each condition is shown in parentheses below the graph; ∼40–100 cells were counted per experiment. (D) Fluorescence intensity ratio between NE and the adjacent cytoplasm in cyclin B1 positive and negative cells (∼50 cells per measurement); cells were either untreated or treated with 10 µM nocodazole for 1 h. Measurements were performed in a region of 0.3 by 30 µm at the border between cytoplasm and the nucleus (NE) and in a directly adjacent region (cytoplasm). Error bars represent SEM. (E) HeLa cells were transfected with a mixture of RanBP2 siRNAs #1 and #2, fixed, and stained as described for (A). Note that the two cyclin B1-positive cells that have strongly reduced RanBP2 levels show no accumulation of BICD2 at the NE.

We next investigated whether endogenous BICD2 and RanBP2 co-localize in HeLa cells and found that BICD2 specifically associates with the NE in a subset of cells, where it largely overlaps with the RanBP2 staining (Figure 2A). This localization pattern was visible not only in cells subjected to fixations with paraformaldehyde or a combination of cold methanol with paraformaldehyde (which are optimal for preservation of the Golgi- and vesicle-bound fraction of BICD2) but also after fixation with cold methanol alone, which did not preserve the Golgi-bound or the cytoplasmic pool of the protein (see below); it was further enhanced by treating cells with the MT-destabilizing drug nocodazole (Figure 2B–D). We hypothesized that the absence of BICD2 staining at the NE in a subset of cells was caused by cell cycle regulation. Indeed, all cells that showed BICD2 accumulation at the NE were positive for cyclin B1, which is expressed exclusively in G2 and mitosis, and ∼75% cyclin B1-positive cells showed BICD2 localization at the NE, indicating that BICD2 associates with the NE in the G2 phase (Figure 2A–D). G2-specific recruitment to the NE was also observed in another human cell line, U2OS cells (Figure S2A,B).

In addition to the NE, endogenous BICD2 also co-localized with RanBP2 in puncta in the cytoplasm (Figure 2A,B). Comparison with previous studies suggested that these puncta are cytoplasmic stacks of NPCs known as annulate lamellae (AL) [39]. Indeed, these puncta were stained by 4 additional markers for NPCs: RanGAP1; the monoclonal antibody 414 (MAB414) that reacts with several nucleoporins [40]; an antibody against NUP214, an NPC component that binds to the cytoplasmic side of the nuclear pores independently of RanBP2 [41], and YFP-tagged POM121, a transmembrane NPC component that is also present in AL (Figure S3) [42]. In line with the recruitment of BICD2 to NPCs in the NE in G2 cells, we observed the association of BICD2 with the AL in cyclin B1-positive but not in cyclin B1-negative cells (Figure 2A,B). These results are important as they support the view that G2-specific recruitment of BICD2 to the NE is due to its interaction with the cytoplasmic part of the NPCs and not some other NE component.

Since we found that BICD2 directly interacts with RanBP2, we examined whether the NE localization of BICD2 was RanBP2-dependent. RanBP2 could be specifically depleted from HeLa and U2OS cells without affecting the expression of BICD2 (Figure S4A,B). Indeed, depletion of RanBP2 blocked recruitment of BICD2 to the NE of G2 cells (Figure 2C,E; Figure S2A,B).

**Figure 3:**
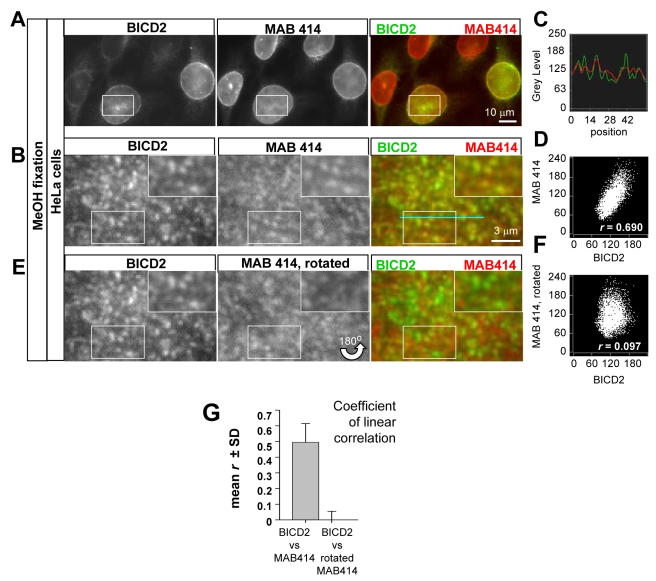
BICD2 to localizes to the nuclear pores at the NE. (A) HeLa cells were fixed with cold methanol and stained with antibodies against BICD2 and nucleoporins (MAB414). These panels show focal planes in the middle part of the nuclei. (B) Enlargements of the flattened NE surface from the same cells as in (A) (the area from which the enlarged image is taken is indicated in (A) by a white rectangle). The insets show further enlargements, in which individual NPC can be distinguished. Colors used for the overlays are indicated above the corresponding images. Note that most NPC dots display signal in both channels, indicating that BICD2 and MAB414 antigens (NPCs) co-localize. (C) Representative fluorescence intensity profile of BICD2 (green) and MAB414 staining (red) at the NE surface. The profile was obtained using Linescan function of MetaMorph at the position indicated by a blue line in (B). Vertical axes are in arbitrary units. Note that most peaks are present in both BICD2 and MAB414 channels, although their intensities often differ, in agreement with the fact that BICD2 is not a nucleoporin. (D) Correlation between BICD2 and MAB414 signals in Figure 3B. The intensity of each pixel in the green and red channel is represented by a dot. (E,F) The same images and analysis as in (B) and (D), but with the MAB414 panel rotated by 180°. Note that there is no co-localization between the green and red signals and no correlation is visible in the plot. (G) Average coefficient of linear correlation between BICD2 and MAB414 signals, determined from plots such as in (B) and (D) obtained for 19 cells. Note that the coefficient is much higher for properly aligned images compared to rotated ones, indicating that co-localization is not spurious. Error bars represent SD.

Based on the results described above, BICD2 is expected to specifically associate with individual NPCs on the NE. Indeed, both full-length BICD2 and GFP-tagged BICD2-CT co-localized with individual NPCs on the NE in cells that were pre-extracted with a Triton X-100-containing buffer to reduce the cytoplasmic pool of the GFP-BICD2 fusions (Figure S5). Similarly, colocalization of endogenous BICD2 with individual NPCs was also observed in methanol-fixed cells, in which cytosolic BICD2 signal has been removed (Figure 3A,B). Fluorescent intensity profiles showed that most of the individual NPCs in the NE (stained with MAB414) also showed a peak of BICD2 fluorescence (Figure 3B,C). The significance of the overlap was confirmed by quantitative analysis: the coefficient of linear correlation between properly aligned MAB414 and BICD2 images was, on average, ∼0.5, while it was close to zero when one of the two images was rotated, indicating that observed colocalization between the two markers was not due to fortuitous overlap between abundant dot-like patterns (Figure 3D–G). Taken together, our results show that BICD2 binds RanBP2 both in vitro and in vivo, localizes to NPCs (both in the NE and in AL) in G2 cells, and that this recruitment to NPCs depends on RanBP2. It is therefore likely that a direct interaction between RanBP2 and BICD2 links BICD2 to the NE in G2 cells.

### BICD2 Recruits the Dynein-Dynactin Complex to the NE and AL during G2

**Figure 4:**
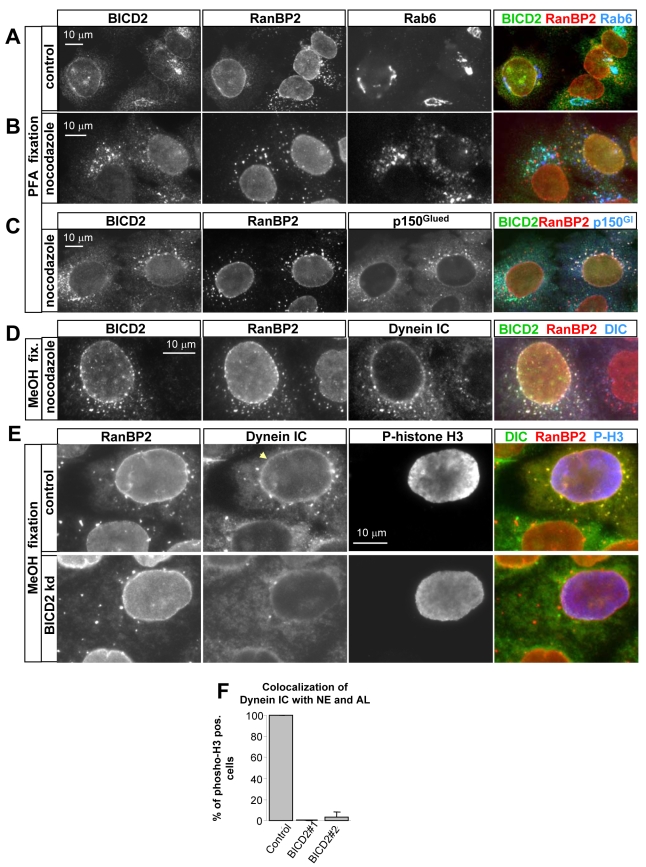
BICD2 is required for targeting dynein/dynactin to the NE and AL. (A–D) Control HeLa cells or HeLa cells treated with 10 µM nocodazole for 1 h were fixed with paraformaldehyde (A–C) or cold methanol (D) and stained for the indicated endogenous proteins. Dynactin is visualized with an antibody to p150^Glued^ and dynein with an antibody to dynein IC. Colors used for the overlays are indicated above the corresponding images. Note that BICD2 shows two types of localization: it colocalizes either with Rab6 or with RanBP2. In cells where BICD2 associates with RanBP2-positive NE and AL, dynein and dynactin are also targeted to these structures. (E) HeLa cells were transfected with a control siRNA (upper panels) or BICD2#1 siRNA (bottom panels). Three days later, cells were treated with 10 µM nocodazole for 5 h, fixed with cold methanol, and stained for endogenous RanBP2, phosphorylated histone H3, and dynein IC. NE staining by dynein antibodies is indicated by an arrow. Colors used for the overlays are indicated above the corresponding images. Note that dynein is enriched at the NE and annulate lamellae in control phospho-histone H3 positive cell, but not in BICD2-knockdown cell. (F) Percentage of HeLa cells positive for phosphorylated histone H3 that show strong accumulation of dynein IC at the RanBP2-positive NE and AL in control or BICD2-depleted cells 3 d after siRNA transfection. Only the cells with clearly visible AL were included in the quantification. Error bars represent SD; ∼25–30 cells were counted in two experiments.

Although previous studies showed that BICD2 strongly co-localizes with Rab6 on the Golgi apparatus and cytoplasmic vesicles [10],[11], this was not the case in G2 cells where BICD2 accumulated at the NE (Figure 4A). This conclusion was confirmed by staining nocodazole-treated cells, where the dispersion of the Golgi and nocodazole-induced enlargement of the AL permitted better distinction of protein localization in different cytoplasmic structures (Figure 4B). In the cells where BICD2 associated with Rab6-bound membranes, it did not stain the NE or the AL. However, in the cells where BICD2 localized to the NE and AL, it displayed virtually no colocalization with Rab6 (Figure 4B). Combined with the results described above, these observations indicate that BICD2 switches from Rab6-bound membranes to the NPCs in G2 phase cells.

We next investigated whether the dynein-dynactin complex is recruited to the NE along with BICD2. In untreated HeLa cells, both dynein and dynactin show diffuse cytosolic localization as well as an accumulation at the MT plus ends and the centrosomes (unpublished data). However, in nocodazole-treated cells, colocalization of dynein and dynactin with BICD2 could be detected in specific cytoplasmic structures [8]. While in the majority of cells these structures coincided with Rab6-positive Golgi fragments, in all cells in which BICD2 co-localized with RanBP2, both dynein and dynactin co-localized with BICD2 on RanBP2-positive membranes, suggesting that the association of BICD2 with dynein and dynactin is maintained when BICD2 switches from Rab6 to RanBP2 (Figure 4C,D; Figure S6A). Because both BICD2 and RanBP2 were shown to bind to kinesin-1, we also attempted to investigate the localization of its isoforms. Although we were able to specifically detect the predominant kinesin-1 isoform, KIF5B, in HeLa cells (as confirmed by siRNA-mediated depletion), it displayed a largely diffuse distribution, and no clear accumulation of this protein could be detected at the NE or AL with or without nocodazole treatment (unpublished data).

Since BICD2 can directly bind both the NPCs and the dynein-dynactin complex, we next investigated whether BICD2 is required to recruit dynein and dynactin to the NPCs. Because we could not stain cells simultaneously for cyclin B1 and dynactin or dynein, we used an antibody against histone H3 phosphorylated at serine 10 (phospho-H3), which becomes highly phosphorylated in late G2/prophase cells [43]. All phospho-H3-positive cells displayed very strong recruitment of endogenous dynactin and dynein to both the NE and AL (Figure 4E,F; Figure S6B,C). Strikingly, this recruitment was inhibited by depletion of BICD2, but not BICD1 (Figure 4E,F; Figure S6B,C; and unpublished data), demonstrating that BICD2 is required for the association of dynein and dynactin with the NPCs in prophase cells. In contrast, BICD2 was recruited normally to the NPCs after depletion of either the dynein heavy chain (HC) or dynactin large subunit p150^Glued^ (see below). These results suggest that BICD2 directly links the dynein/dynactin complex to the NE through its interaction with RanBP2.

### Dynein and Kinesin-1 Control Relative Positioning of the Nucleus and the Centrosomes before Mitotic Entry

**Figure 5:**
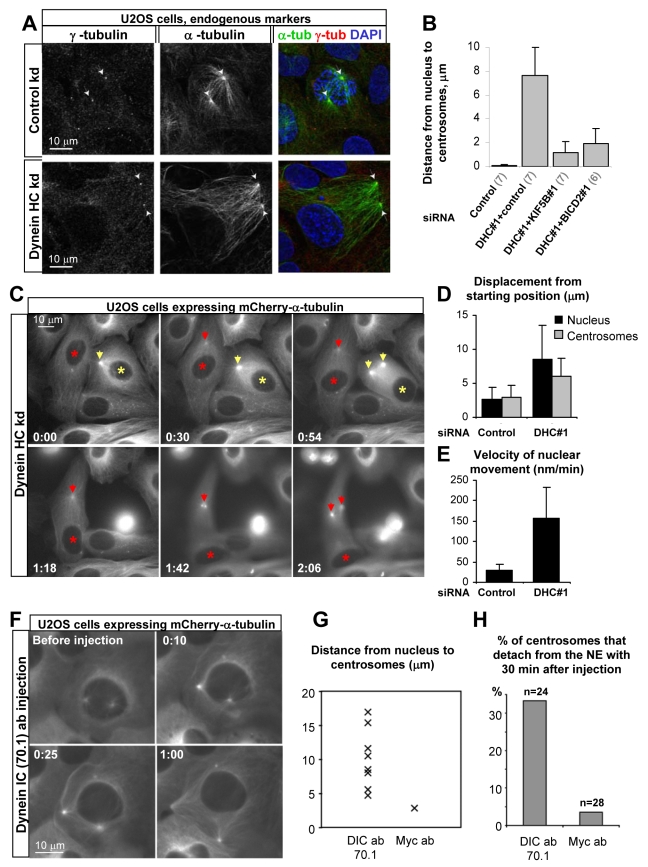
Dynein inactivation causes rapid separation of nuclei and centrosomes in G2. (A) U2OS cells were transfected with the control or DHC#3 siRNAs, fixed with cold methanol, and stained for α-tubulin (green in overlay) and γ-tubulin (red in overlay), as well as DNA (DAPI, blue in overlay). Note that centrosomes are located close to the nucleus in control cells but are removed far away from the nucleus in dynein-depleted cells. (B) mCherry-α-tubulin stable U2OS cell line was imaged with a 3 min time interval 2.5 d after transfection with the indicated siRNA mixtures, and the distance between centrosomes and the nucleus at the time of NEB was measured. Error bars represent SD. The number of experiments is indicated in parentheses below the graph. ∼20 cells per experiment were analyzed. (C) mCherry-α-tubulin stable U2OS cells were imaged with a 3 min interval 2.5 d after transfection with DHC#1 siRNA. Nuclei and centrosomes undergo G2-specific displacements (indicated by asterisks and arrows, respectively), resulting in their separation. (D,E) Quantification of displacement of nuclei and centrosomes starting from the moment when G2-specific enhanced nucleation of MTs at the centrosome became visible (D), and velocity of nuclear movement during 1 h before NEB (E). Nuclear movement in dynein-depleted cells was initiated 54±4 min (mean±SD) before NEB. Measurements were performed in 10 cells in 3 independent experiments. (F–H) U2OS cells stably expressing mCherry-α-tubulin were microinjected with antibodies against dynein IC (70.1) or the Myc tag. Late G2 cells were chosen based on the presence of separated centrosomes. (F) An example of nucleus-centrosome separation after microinjection with dynein-inhibiting antibodies. (G,H) Distance between the nucleus and the centrosomes (G) and percentage of cells showing strong centrosome detachment (H) at 30 min after microinjection.

Since dynein associates with the NE in G2 phase, we next examined how its depletion affected the relative position of the nucleus and centrosomes during mitotic entry. Because the perinuclear MT cytoskeleton is very dense and therefore difficult to analyze in HeLa cells, we used U2OS cells, in which MT arrays are more sparse and centrosome-centered. In control cells, the centrosomes were always located very closely to the NE in prophase (Figure 5A,B). In contrast, in dynein-depleted cells the nucleus and the centrosomes were almost always found in opposite cell corners during prophase (Figure 5A,B). Similarly, live cell imaging of U2OS cells stably expressing mCherry-α-tubulin showed that in control cells centrosomes migrate along the NE, to the opposite sides of the nucleus just before mitotic entry (Figure S7A), allowing spindle assembly to initiate around the DNA. In contrast, in ∼90% of dynein-depleted cells, centrosomes and the nucleus had moved apart substantially at the time NEB occurred, and therefore the DNA was not positioned in between the centrosomes when spindle assembly initiated (Figure S7A; Figure 5B); similar results were obtained in HeLa cells after dynein or dynactin depletion (Figure S7B, Videos S1–S3).

Importantly, not only the centrosomes lost their central position (which could be explained by the loss of interactions between MT ends and the cell cortex upon dynein depletion [44]), but also the nucleus was rapidly pushed into one of the cell corners, towards MT plus ends (Figure 5C–E, see Videos S4–S7). Nuclear movement was initiated 54±4 min before NEB, and the average velocity of movement was ∼160 nm/min (Figure 5E). The final distance between the two centrosomes at the time of NEB was the same in control and dynein-depleted cells (9.8±1.0 µm in control and 9.8±0.6 µm in dynein-depleted cells (mean±SD)), consistent with our previous findings [45], indicating that centrosome separation does not require linkage to the NE or dynein function.

To rule out that the detachment of centrosomes from the nucleus was due to defects arising after long-term dynein inhibition, we microinjected U2OS cells that were in late G2 with function blocking antibodies against dynein intermediate chain (IC). As expected, dynein-inhibiting antibodies, but not the control antibodies, induced rapid separation of the nuclei and the centrosomes (Figure 5F–H), demonstrating that dynein activity is required during late G2 to maintain the connection between centrosomes and the NE. Very similar results were obtained after microinjection of the first coiled coil fragment of the dynactin large subunit p150^Glued^ (CC1), which is known to disrupt dynactin-dependent dynein mediated processes (Figure S8) [46].

Pushing a relatively large nucleus into a flattened corner of a cultured cell would require substantial force, which is most likely generated by kinesin motors attached to the nucleus and moving to MT plus ends. We hypothesized that KIF5B might be involved in this process because it interacts with both BICD2 and RanBP2. Indeed, co-depletion of KIF5B together with dynein fully restored centrosome and nuclear position at NEB (Figure 5B, Figure S7A; for control of double knockdown efficiency, see Figure S4C), indicating that it is indeed driving the separation of nuclei and centrosomes in dynein-depleted cells. Taken together, these results suggest that the prophase cell nucleus is transported bi-directionally by the opposing activities of dynein and kinesin-1, similar to many other cargoes.

### BICD2 and RanBP2 Are Required for Maintenance of the Association between the NE and the Centrosomes in Prophase Cells

**Figure 6:**
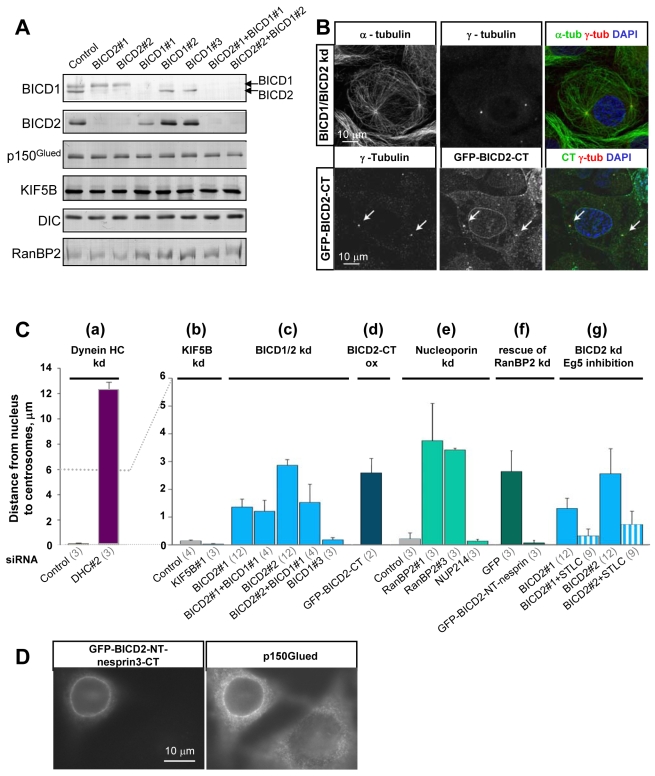
Depletion of BICD2 and RanBP2 causes centrosome detachment in prophase U2OS cells. (A) Western blots with the indicated antibodies were performed with equal amounts of extracts of U2OS cells 3 d after transfection with the indicated siRNAs. Note that BICD1 antibody cross-reacts with BICD2 (arrows). Both BICD proteins can be independently depleted, although BICD1#1 siRNA causes some co-depletion of BICD2. Levels of dynactin, dynein, KIF5B, and RanBP2 are not significantly affected BICD knockdown. (B) U2OS cells were transfected with BICD2#1 and BICD1#1 siRNAs (upper panel) or with GFP-BICD2-CT, fixed with cold methanol, and stained for α-tubulin and γ-tubulin, as well as DNA (DAPI). Colors used for the overlays are indicated above the corresponding images. Note centrosome detachment caused by the loss of BICD function through siRNA-mediated depletion or overexpression of the dominant negative mutant BICD2-CT. (C) The distance between the nucleus and the centrosomes in fixed prophase U2OS cells under different conditions. (a,b) Cells were transfected with the indicated siRNAs against dynein HC or KIF5B. (c) Cells were transfected with the indicated siRNAs against BICD1 and/or BICD2. (d) Cells were transfected with GFP-BICD2-CT. (e) Cells were transfected with the indicated siRNAs against RanBP2 or NUP214. (f) The effect of RanBP2 knockdown (induced with the RanBP2#3 siRNA) was rescued by expression of the GFP-BICD2-NT-nesprin-3 fusion but not by GFP alone. (g) Cells were transfected with the indicated siRNAs against BICD2, and either untreated or incubated with 4 µM STLC, an Eg5 inhibitor. Error bars represent SD; the number of experiments for each condition is shown in parentheses below the graph; 20–30 cells were counted per experiment. Strong separation of the nucleus and the centrosomes is induced by dynein depletion. Mild nuclear-centrosome separation is caused by overexpression of BICD2-CT or depletion of BICD2 or RanBP2, but not BICD1 or NUP214, which serve as negative controls. A fusion of BICD2 N-terminus to the NE-targeting domain can compensate for the loss of RanBP2. The effect of BICD2 knockdown on nuclear-centrosome positioning can be suppressed by inhibiting Eg5, suggesting that the MT sliding activity of this motor is responsible for centrosome detachment in BICD2-depleted cells. (D) HeLa cells were transfected with GFP-BICD2-NT-nesprin-3 fusion, fixed with paraformaldehyde, and stained for p150^Glued^. Note the recruitment of dynactin to the NE in the transfected cell.

Since we showed that BICD2 is required for dynein and dynactin recruitment to the NPCs in G2 phase, we next investigated whether its depletion has an influence on the relative positioning of the centrosomes and the nuclei. Similar to HeLa, U2OS cells express both BICD2 and BICD1, which can be depleted by a number of different siRNAs without affecting the expression of RanBP2 or MT motors (Figure 6A; Figure S4C).

Depletion of BICD2 induced the detachment of the centrosomes from the nucleus in prophase (Figure 6B,C(a–c)). This effect was specific, since it was not observed after depletion of BICD1 or KIF5B, but it was much less severe than after dynein knockdown (Figure 6B,C(a–c)). Cells with simultaneous knockdown of BICD1 and BICD2 displayed a phenotype similar to that of BICD2 depletion alone, confirming the view that BICD1 does not contribute much to centrosome positioning (Figure 6C(c)). Centrosome detachment from the NE was also observed in cells overexpressing BICD2-CT, which is expected to uncouple dynein/dynactin from BICD2-containing cargos (Figure 6B,C(d)) [11]. Interestingly, RanBP2 depletion, which prevents BICD2 recruitment to the NE (Figure 2C,E; Figure S2A,B) also caused an increase in the distance between the nuclei and the centrosomes, while the depletion of another cytoplasmic nucleoporin, NUP214, had no effect (Figure 6C(e); Figure S4B).

To further prove that BICD2 can exert a direct effect on centrosome positioning through its localization at the NE, we constructed a fusion protein in which we attached the N-terminal portion of BICD2, including the dynein and kinesin-1 binding sites, to the C-terminal KASH (Klarsicht, ANC-1, Syne Homology) domain-containing region of nesprin-3, which is targeted to the NE by SUN proteins [47]. This fusion localized specifically to the NE and enhanced the accumulation of dynactin at the NE (Figure 6D). Importantly, the expression of the BICD2-NT-nesprin-3 fusion completely suppressed centrosome detachment in RanBP2-depleted prophase cells (in which endogenous BICD2 is no longer targeted to the NE, see Figure 2C,E), even at very low expression levels (Figure 6C(f)). Taken together, these results support the view that BICD2 can recruit MT motors to the NE and regulate the relative localization of the nucleus and the centrosomes.

Since the distance between the centrosomes and the nucleus in BICD2 or RanBP2 knockdown cells was much smaller than in the case of dynein knockdown, it appears that there is no severe imbalance between the activities of dynein and kinesin-1 at the NE under these conditions. These results suggest that BICD2 is not only involved in dynein function at the NE but might also be required for proper kinesin-1 function.

If this idea is correct, BICD2 depletion should block the kinesin-1 driven separation of centrosomes from the nuclei observed after dynein depletion. Indeed, co-depletion of BICD2 with dynein strongly reduced the distance between the centrosomes and the nuclei observed after dynein depletion alone (see Figure 5B). This rescue of centrosome-nuclear attachment was not due to a decreased efficiency of dynein depletion (see Figure S4C); moreover, the mitotic arrest in cells depleted of both BICD2 and dynein HC was similar to that observed for single dynein HC knockdown (unpublished data), further confirming that dynein function was similarly perturbed in both cases and indicating that BICD2 depletion does not help to overcome later mitotic phenotypes associated with dynein loss.

### Eg5 Pushes the Centrosomes Away from the Nucleus in BICD2-Depleted Cells

Why do centrosomes detach from the NE in prophase after BICD2 knockdown if both kinesin-1 and dynein activities are reduced? We recently found that the plus-end directed kinesin-5 Eg5, known to slide antiparallel MTs [48], pushes centrosomes apart during prophase [45]. Thus, Eg5-dependent sliding forces might drive the centrosomes away from the nucleus when it becomes uncoupled from dynein and kinesin-1 due to BICD2 depletion. In line with this idea, inhibition of Eg5 with S-trityl-L-cysteine (STLC) significantly suppressed centrosome detachment caused by BICD2 depletion, indicating that Eg5 is at least in part responsible for pushing the centrosomes away from NE in BICD2-depleted prophase cells (Figure 6C(g)). Taken together, our data show that centrosome separation and positioning at the opposite sides of the NE at the mitotic onset is driven by forces generated by dynein, kinesin-1, and Eg5.

### G2-Specific Movement of AL Confirms the BICD2-Dependent Link between NPCs and MT Motors

**Figure 7:**
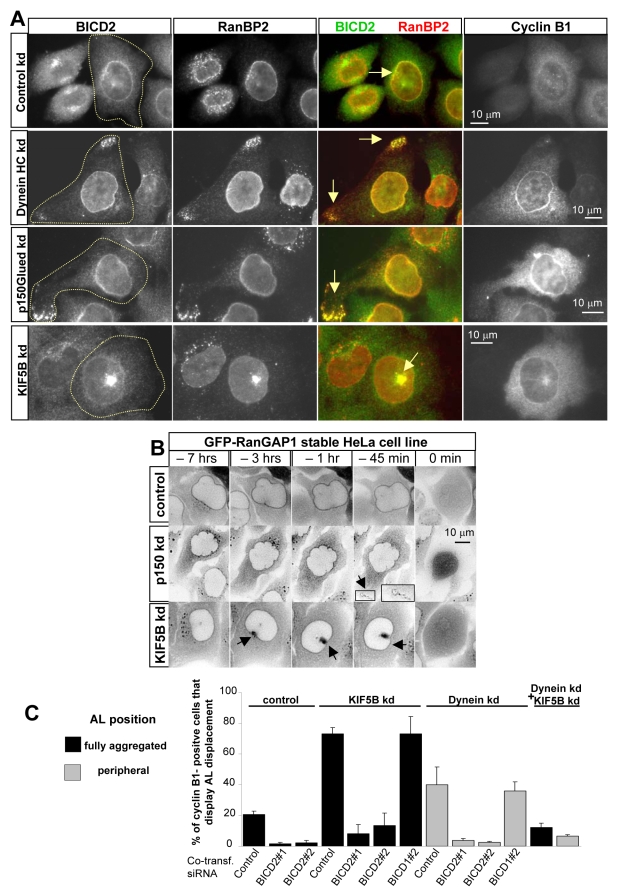
Dynein, kinesin-1, and BICD2 control the localization of AL in G2 phase. (A) HeLa cells were transfected with control, DHC#1, p150^Glued^, or KIF5B#1 siRNAs, fixed with paraformaldehyde 3 d later, and stained for endogenous BICD2, RanBP2, and cyclin B1. In the overlays, BICD2 is shown in green and RanBP2 in red. The outline of the cyclin B1-positive cell is indicated. Note AL displacement to the cell periphery in cyclin B1-positive cells depleted of dynein or dynactin, and the centripetal relocalization of AL in kinesin-1 (KIF5B) depleted cyclin B1-stained cell. (B) GFP-RanGAP1 stable HeLa cell line was imaged with a 2 or 3 min time interval 2 d after transfection with the control, p150^Glued^, or KIF5B#1 siRNAs. 0 min indicates the first frame after NEB (defined as the time when GFP-RanGAP1 enters the nucleus). Contrast is inverted. Arrows show the accumulation of AL at the cell periphery or the cell center. Peripheral displacement of AL after dynein or dynactin knockdown occurred at 1 h±30 min before mitotic onset (mean ± SD, measured in 6 and 10 cells, respectively); strong accumulation of AL near the nucleus in KIF5B depleted cells started at 3 h±30 min before NEB (mean ± SD, measured in 18 cells). (C) AL localization after different siRNA co-transfections. Black bars represent the percentage of HeLa cells showing a strong AL accumulation near the nucleus (like in the bottom panel in Figure 7A) and gray bars illustrate the percentage of HeLa cells showing displacement of AL to the outmost cell periphery (like in the middle panels of Figure 7A). In each case, cells were transfected with a combination of two siRNAs: control, KIF5B or dynein HC siRNAs (as indicated on top of the panel) in combination with the control siRNA or the siRNAs against BICD1 and BICD2 (the cotransfected siRNA is indicated at the bottom), or the combination of KIF5B siRNAs and dynein HC siRNAs. Cells with AL accumulation in the cell center or the cell periphery were scored in KIF5B or dynein knockdown cells, respectively; in a double KIF5B/dynein HC knockdown, both types of cells were counted. Error bars represent SD. ∼30–100 cells were counted in three experiments. Cells with fully aggregated AL represent a minority of the population (∼20%) in control G2 cells but become predominant (∼75%) after depletion of kinesin-1. This effect can be suppressed by co-depletion of BICD2 or dynein HC, but not BICD1. Similarly, control cells never display peripheral AL localization, while ∼40% of dynein-depleted cyclin B1-positive cells show this phenotype, which can be suppressed by co-depletion of BICD2 or KIF5B, but not BICD1.

Do dynein and kinesin-1 indeed attach to the NE through NPCs? If this were true, the positioning of AL, which contain cytoplasmic NPC components but are devoid of many other NE-specific proteins, such as the nuclear lamina and the proteins that are linked to it, should be affected by the depletion of MT motors. AL are relatively small structures that are normally located in the central cytoplasm; they can potentially serve as a sensitive readout for the forces exerted on them by cytoplasmic motors. In control cells, AL are predominantly located around the Golgi apparatus in G1 and S-phase; in G2 they shift towards the centrosome and gradually disappear ([49], Figure 7A, and see also Figures 2A, 4A, and Video S8). The depletion of the dynein HC or dynactin subunit p150^Glued^ induced relocalization of AL to the cell periphery of cyclin B1-positive cells (towards MT plus ends; Figure 7A). Using a cell line stably expressing GFP-tagged RanGAP1 (Figure S9), we observed that the timing of movement of the AL to the cell periphery coincided almost exactly with the timing of peripheral displacement of the nucleus in cells lacking dynein activity (∼1 h before NEB) (Figure 7B, Video S9). Furthermore, peripheral displacement of AL in dynein-depleted cells was completely dependent on kinesin-1 activity (Figure 7C), similar to displacement of the nucleus in these cells (see Figure 5B). Together, these results indicate that the forces that act to position the AL are mechanistically similar to those that position the nucleus. Consistent with this, knockdown of KIF5B caused a very strong accumulation of AL near centrosomes, where MT minus ends are located in G2 (Figure 7A and Figure S10A,B,C), and centrosomal accumulation of AL was, in turn, dependent on dynein activity (Figure 7C). The analysis of the timing of AL movement in kinesin-1-depleted cells showed that centrosomal accumulation started ∼3 h before NEB (Figure 7B, Video S10), which corresponds very well to the time at which BICD2 and dynein are recruited to NPCs, further suggesting that BICD2 and dynein recruitment to NPCs in G2 phase induces movement of these structures towards MT minus ends. Taken together, these results show that, similar to the nucleus, the position of AL in G2 phase is controlled by the antagonistic activities of dynein and kinesin-1 and further suggest that both dynein and kinesin-1 act to position the nucleus through their effect on NPCs, rather than through other NE-associated proteins.

We also used AL displacement to strengthen our conclusions that BICD2 controls both dynein- and kinesin-1-dependent movement of NPCs. Importantly, BICD2 remained strongly enriched at the AL and the NE when dynein, dynactin, or KIF5B were depleted, indicating that BICD2 association with the NPCs is independent of MT motors (Figure 7A). In control G2 phase cells, AL often displayed some perinuclear accumulation in the centrosome region (see Figures 2A, 7C, Figure S11A,B). However, BICD2 depletion reduced perinuclear accumulation of AL in G2 phase (Figure 7C, Figure S11A,B). These findings were confirmed by observing the behavior of AL by time-lapse microscopy after BICD2 knockdown, where AL remained randomly dispersed until the beginning of mitosis (Figure S11C, Video S11). These results further implicate BICD2 in the G2 specific activation of MT minus-end-directed force generation on NPCs. Furthermore, the shift of AL to the cell center or the cell periphery caused by kinesin-1 and dynein depletion, respectively, was strongly inhibited by co-depletion of BICD2 but not by control siRNAs or siRNAs against BICD1 or Rab6 (Figure 7C, Figures S11C, S12; Videos S12, S13). These results demonstrate that BICD2 is required for the G2-specific movement of the AL by both kinesin-1 and dynein and strongly support the notion that both dynein and kinesin-1 control nuclear movement by acting through BICD2 on the NPCs.

We have also investigated if other cytoskeletal systems, in addition to MTs, are directly involved in the G2-specific processes described above but found no evidence for direct involvement of the actin cytoskeleton or the intermediate filaments, keratin or vimentin, in the G2-specific nuclear-centrosome positioning pathway that relies on dynein and kinesin-1 (Figures S13, S14).

## Discussion

During cell division the MT cytoskeleton and membrane organelles undergo a severe reorganization, which proceeds in a highly regulated manner. In many cell types, the two centrosomes move apart while maintaining their attachment to the NE. This helps to form the bipolar mitotic spindle around the chromosomes after NEB. In this study, we have obtained insight into molecular mechanisms that control the relative positioning of the nucleus and the centrosomes at mitotic onset. We show that the dynein/dynactin adaptor BICD2 is specifically recruited to the NPC in G2 phase through a direct interaction with the NPC component RanBP2. In its turn, BICD2 is important for accumulation of dynein and dynactin at the nuclear pores in prophase cells. In line with previously published data (reviewed by [27],[28]), we find that cytoplasmic dynein is the major player responsible for the nucleus-centrosome attachment, but unexpectedly, we find that kinesin-1 also participates in this process by antagonizing dynein function. Since BICD2 and RanBP2 are likely involved in linking both MT motors to the NPCs, depletion of either protein causes only mild centrosome detachment from the nucleus.

**Figure 8:**
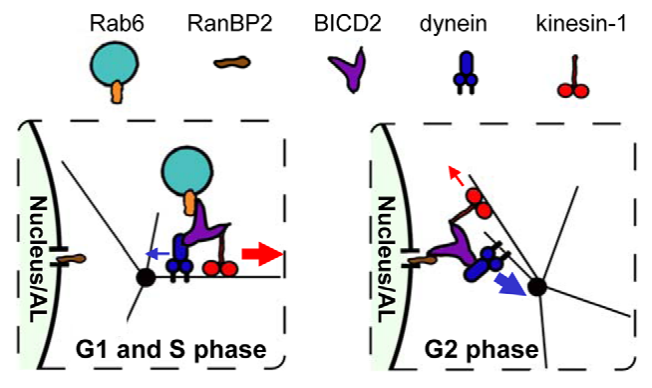
A model of the concerted action of BICD2, dynein, and kinesin-1 in different phases of the cell cycle. In G1 and S phase, BICD2, dynein, and kinesin-1 associate with Rab6 vesicles; kinesin-1 activity predominates in this complex. In G2, BICD2 with the associated motors accumulates at the NPCs, where the dynein-mediated movement predominates, resulting in tight association of the centrosome and nucleus.

Our previous studies showed that BICD2 associates with MT motors through its N-terminus and the middle portion, while the C terminus is the cargo-binding site [9],[11],[13]. Here we identified a new cargo for BICD2, the nucleoporin RanBP2, which binds to the same domain of BICD2 as the small GTPase Rab6. Our data suggest that the interaction of BICD2 with the two cargos is temporally regulated during the cell cycle: during G1 and S phase, BICD2 appears to associate predominantly with Rab6, while in G2 it binds mostly to the NPCs (Figure 8). It is currently unclear how this switch is controlled, but it is likely that mitotic kinases are involved.

Both during Rab6 vesicle trafficking and in nuclear positioning, BICD2 participates in transport processes that involve the opposing functions of cytoplasmic dynein and kinesin-1. The predominating motor in the two processes is different: Rab6 vesicles are exocytotic carriers that preferentially move to MT plus ends, suggesting that kinesin-1 activity is dominant, while the nucleus and AL are mainly pulled by dynein (Figure 8). This indicates that BICD2 participation by itself is insufficient to determine direction of movement; therefore, additional factors or posttranslational modifications are likely to be involved. While it may appear strange that the two opposite polarity motors act together in processes that mostly depend on only one of them, this arrangement seems to represent a fundamental property of MT motor systems most likely required to allow flexibility and permit regulation of cargo distribution [3],[4]. Our study shows that even the positioning of a very large cargo, such as the cell nucleus, is no exception to this rule.

The mechanism underlying kinesin-1 recruitment to BICD2-bound NPCs is unlikely to be explained solely by the binding between BICD2 and kinesin-1 [13], since RanBP2 can directly bind to kinesin-1 as well [36],[37]. Intriguingly, both BICD2 and kinesin-1 interact with the same region of RanBP2; whether these interactions are competitive or cooperative and what consequences this has on the architecture of the motor complexes remains to be determined.

It is clear that RanBP2 and BICD2 are not the only proteins participating in the motor recruitment and/or activation important for centrosome-nuclear attachment. First, other parts of the NPCs, additional dynein accessory factors, such as LIS1 and NUDE [50], and cell cycle-dependent regulators of motor activation, like CDKs or Plk1, are likely to be involved. This view is supported by the observed timing of binding and transport steps. BICD2 associates with the NPCs early in G2; this results in dynein activation that is sufficient to cause strong AL accumulation around the centrosome in the absence of kinesin-1 at ∼3 h before NEB. At a later stage (∼1 h before NEB), additional motor activation likely takes place; this is reflected by the peripheral displacement of the AL, the nucleus, and the centrosomes in dynein-depleted cells. Furthermore, Eg5 becomes active during prophase and pushes centrosomes apart. The forces induced by Eg5-dependent centrosome separation are kept in check by the complex of RanBP2-BICD2-dynein that prevents centrosome detachment from the nucleus while allowing centrosomes to separate.

Second, KASH domain proteins such as nesprins are essential for attachment of nuclei to the cytoskeleton in different systems [33],[51],[52]. We observed that the displacement of endogenous nesprins from the NE indeed affected relative positioning of the nucleus and centrosomes in G2 phase, but the effect was much less severe than that of dynein depletion (Tanenbaum, unpublished data). Moreover, nesprin displacement from the NE could not block kinesin-1-mediated nuclear displacement in dynein-depleted cells (Splinter, unpublished data), indicating that in contrast to certain epithelial cells [53], in cultured U2OS and HeLa cells nesprins are not essential for attachment of kinesin-1 to the NE. Nesprins bind to the NE through SUN proteins, which were shown to interact with nuclear pores [54], and may therefore participate in the formation of the MT motor assemblies at the NE together with nucleoporins. It is likely that the relative importance of different molecular links between the NE and MT motors depends on the cell type and differentiation state.

What is the function of the complex molecular events described in this study? Clearly, positioning of the centrosomes at the opposite sides of the nucleus at NEB would decrease the chance that a kinetochore is captured by MTs emanating from both poles (merotelic attachments), which can result in chromosome missegregation and aneuploidy, hallmarks of cancer [55]. Furthermore, spindle assembly in mammalian cells is controlled by both centrosome- and chromatin-dependent pathways, in which centrosomes are potent MT nucleation sites and chromatin can both nucleate and stabilize MTs. When centrosomes are positioned too far away from chromatin, newly nucleated MTs are unstable and as a consequence spindle assembly might be delayed. Indeed, loss of dynein results in a ∼15 min delay in bipolar spindle assembly, which can be rescued by restoring the relative positioning of centrosomes and the nucleus at mitotic entry through co-depletion of kinesin-1 (Tanenbaum, unpublished data). A mechanism coupling centrosomes to the nucleus at mitotic onset could become even more important in very large cells like fertilized oocytes, in which the distance between centrosomes and chromosomes could become so extensive that centrosomes could no longer contribute to spindle assembly.

In addition, the interaction of dynein with the NPCs through BICD2 could help to tear apart the NE [26],[56], a possibility that was not addressed here. Furthermore, dynein-mediated coupling between the nucleus, MTs, and the centrosome plays an important role during migration of differentiated cells [57]. In flies, BicD is involved in MT and dynein/dynactin-dependent positioning of the oocyte and photoreceptor nuclei [7], and since BICD2 is ubiquitously expressed during mammalian development (Akhmanova, unpublished data), it would be interesting to know if it plays a similar role in mammals.

## Materials and Methods

### Expression Constructs and siRNAs

We used the following previously described expression vectors: GFP-BICD2 [8], HA-BICD2-CT [11], myc-KIF5B [13], BirA [58] (a gift of D. Meijer, Erasmus MC, Rotterdam, The Netherlands), mCherry-α-tubulin [59] (a gift of R. Tsien, UCSD, San Diego, CA, USA). Biotinylation and GFP-tagged BICD2 C terminus (Bio-GFP-BICD2-CT, BICD2 amino acids 487–820, accession number CAC51393) was generated in pEGFP-C2 (Clontech) by cloning at the NheI and AgeI sites in front of the GFP a linker encoding the amino acid sequence MASGLNDIFEAQKIEWHEGGG. CFP-tagged RanBP2 fragments with the N-terminal palmitoylation signal derived from GAP-43 were generated in a modified version of the pECFP-N1 vector (Clontech) by a PCR based strategy. GFP-BICD2-NT-nesprin-3 fusion was generated by attaching the amino acids 582–975 of nesprin-3 (accession number NP_001036164, [60]; a gift of A. Sonnenberg, Netherlands Cancer Institute, Amsterdam) to the C terminus of GFP-BICD2-NT (amino acids 1–594 of BICD2 [9]). GFP-RanGAP1 was generated in pEGFP-C1 by inserting into it the BglII-SmaI fragment of KIAA1835 (accession number AB058738, a gift of Kazusa DNA Research Institute, Japan). POM121 fused to a triple YFP tag [42] was a gift of Dr. E. Hallberg (Södertörns University College, Huddinge, Sweden).

We used the following siRNAs: KIF5B#1, 5′-GCCUUAUGCAUUUGAUCGG (siRNA 118426, Ambion), KIF5B#2, 5′-GCACAUCUCAAGAGCAAGU (siRNA 118427, Ambion), dynein HC DHC#1 5′-CGUACUCCCGUGAUUGAUG (siRNA 118309, Ambion), DHC#2 5′-GCCAAAAGUUACAGACUUU (siRNA 118311, Ambion), DHC#3 5′-GGAUCAAACAUGACGGAAU, RanBP2#1 5′-GGACAGUGGGAUUGUAGUG [61], RanBP2#2 5′-CACAGACAAAGCCGUUGAA, RanBP2#3 Dharmacon SMARTpool, p150^Glued^ 5′ GUAUUUGAAGAUGGAGCAG, BICD2#1 5′-GGAGCUGUCACACUACAUG, BICD2#2 5′-GGUGGACUAUGAGGCUAUC, BICD1#1 5′-CCUUAAUGCCAUAAUCCGG, BICD1#2 5′-GCAAAGAGCCAAUGAAUAU, BICD1#3 5′-GCAACUGUCUCGUCAAAGA, NUP214 5′ GUCACGGAAACAGUGAAAG [41]. As a control we used a previously described scrambled CLASP1 siRNA, the siRNA against luciferase [62], or the siRNA to GAPD (control Dharmacon SMARTpool).

### Pull-Downs, IP, Identification of BICD2-CT Binding Partners by Mass Spectrometry, and Yeast Two-Hybrid Analysis

Bio-GFP-BICD2-CT and BirA were transiently co-expressed in HeLa cells; cells were lysed in a buffer containing 100 mM NaCl, 20 mM Tris-HCl, pH 7.5, 1% Triton X-100, and protease inhibitors (Complete, Roche). Streptavidin pull-down assays, mass spectrometry analysis, and IP from HEK293 cells overexpressing different protein fusions were performed as described by [13]. For the IP of endogenous proteins, HeLa cells were pelleted and lyzed in a buffer containing 20 mM Tris pH 8.0, 150 mM KCl, 5% Triton X-100, Protease inhibitors (Complete Roche) and phosphatase inhibitors (Cocktail 1 and 2, Sigma). For the IP of endogenous proteins from nocodazole-arrested cells, HeLa cells were treated with 75 ng/mL nocodazole for 18 h, washed with PBS, lyzed with digitonin in the buffer containing 20 mM HEPES pH 7.3, 110 mM potassium acetate, 2 mM magnesium acetate, 1 mM EGTA, 1 mM DTT and protease and phosphatase inhibitors; lysates were centrifuged at 100000×g for 1 h. IP was carried out using standard procedures. 6XHIS-tagged BICD2-CT (amino acids 630–820) was generated in pET28a. GST fusions of RanBP2 fragments 3 and 4 (amino acids 2147–2287 and 2447–2887, accession number NP_006258) were generated in pGEX-3X. Protein purification and GST pull-down assays were carried out as described by [63]. Binding reactions were performed in 50 mM Tris-HCl, pH 7.5, 125 mM NaCl, 0.1% NP40, and 5 mM EDTA, using ∼20 µg/ml of HIS-BICD2-CT and ∼70 µg/ml of GST fusions.

For yeast two-hybrid assays, different bait constructs were prepared in pBHA (lexA fusion vector) and tested against various BICD2 fragments cloned into pGAD10 (GAL4 activation domain vector, Clontech) as described by [13].

### Cell Culture and Transfection of Plasmids and siRNAs

HeLa, HEK293, and U2OS cells were cultured as described previously [64],[65]. PolyFect (Qiagen), Lipofectamine 2000 (Invitrogen), or FuGENE 6 (Roche) reagents were used for plasmid transfection. Stable HeLa clones expressing fluorescent proteins were selected using Fluorescence Activated Cell Sorting and cultured in the presence of 0.4 mg/ml G418 (Roche). Synthetic siRNAs were transfected into HeLa cells plated at 20% confluence using HiPerFect (Qiagen) at the final concentration 5 nM; cells were analyzed by 3 d after transfection. U2OS cells were transfected with HiPerFect during plating at ∼20% confluence using 20 nM siRNAs; a second transfection with the same siRNA concentration was performed 1 or 2 d later, and the cells were analyzed 3 or 4 d after plating.

### Antibodies, Immunofluorescent Staining, and Western Blotting

We used affinity purified goat polyclonal antibodies against RanBP2 and RanGAP1 [22],[66]; rabbit polyclonal antibodies against GFP (Abcam), BICD1 and BICD2 [8],[11], HA tag, dynein HC, KIF5B and RanGAP1 (Santa Cruz), NUP214 [41] (a gift of Dr. R. Kehlenbach, University of Göttingen, Germany), and phosphorylated histone H3 (Ser 10) (Millipore); mouse monoclonal antibodies against Rab6 (which recognizes Rab6A and Rab6A', a gift of A. Barnekow, University of Muenster, Germany), Arp1 (a gift of Dr. T Schroer, Johns Hopkins University, USA), nucleoporins (antibody 414, Covance), α-, β-, and γ-tubulin (Sigma), dynein IC (Chemicon and Santa Cruz), cyclin B1 (Santa Cruz), p150^Glued^ and p50 (BD Biosciences), pan-keratin (clone C11, Sigma), and vimentin (Cymbus Biotechnology). For secondary antibodies we used Alexa 350, Alexa 488, and Alexa 594-conjugated goat antibodies against rabbit, rat, and mouse IgG, donkey antibodies against sheep IgG (Molecular Probes), AMCA-labeled rat anti-mouse, FITC-labeled donkey anti-rabbit, and anti-mouse antibodies (Jackson ImmunoResearch Laboratories). Actin was stained with Alexa-594-conjugated phalloidin (Invitrogen). Cell fixation and staining procedures were described previously [8]. Briefly, we used the following fixations: 4% paraformaldehyde in PBS (15 min at room temperature), −20°C methanol (10 min), or −20°C methanol (10 min) immediately followed by 4% paraformaldehyde in PBS (15 min at room temperature). For pre-extraction of live cells we used the following buffer: 60 mM PIPES, 25 mM HEPES, 10 mM EGTA, 0.5% Triton X-100, 4 mM MgSO_4_, and pH 7.5. Western blotting was performed as described previously [64].

### Electron Microscopy

For preembedding immunoperoxidase electron microscopy, cells were fixed with 4% paraformaldehyde and stained with anti-RanGAP1 using the same conditions as for immunofluorescence with the exception that saponin (0.1%) was used as a detergent in the preincubation step and no detergent was used in subsequent steps. Biotinylated horse anti-goat (Vector) was used as the secondary antibody, which was followed by incubation in avidin-biotin-peroxidase complex (ABC, Vector Laboratories, USA) and staining with diaminobenzidine (DAB, 0.05%) yielding a brown reaction product. Subsequently cells were fixed in 1% osmium, dehydrated, and embedded in Durcupan. Ultrathin (50–70 nm) were contrasted with uranyl acetate and lead citrate and analyzed in a Phillips CM100 electron microscope with a bottom mounted TVIPS FastScan-F114 camera.

### Fluorescence Microscopy and Image Analysis

Images of fixed cells with the exception of Figures 5A and 6B were collected with a Leica DMRBE microscope equipped with a PL Fluotar 100× 1.3 N.A. or 40× 1.00–0.50 N.A. oil objectives, FITC/EGFP filter 41012 (Chroma) and Texas Red filter 41004 (Chroma), and an ORCA-ER-1394 CCD camera (Hamamatsu). Images in Figures 5A and 6B were acquired on a confocal Zeiss LSM510 META (CarlZeiss) with a Plan Apochromat 63× 1.4 N.A. objective. Z-planes were acquired with 1 µm intervals. Images are maximum intensity projections of all Z-planes.

Live cell imaging experiments with U2OS cells were performed on a Zeiss Axiovert 200 M microscope equipped with a Plan-Neofluar 40× 1.3 N.A. oil objective in a permanently heated chamber with 5% CO_2_. Images were acquired every 3–5 min using a Photometrics Coolsnap HQ charged-coupled device camera (Scientific, Tucson, AZ). *Z*-stacks were acquired with 2 µm intervals between *Z*-slices. Live cell imaging experiments with HeLa cells were performed on the inverted microscope Nikon Eclipse TE2000E (Nikon) with a CFI Plan Fluor 40× 1.30 N.A. oil objective, equipped with CoolSNAP-HQ2 CCD camera (Roper Scientific) controlled by MetaMorph 7.1 software (Molecular Devices). For excitation we used HBO 103 W/2 Mercury Short Arc Lamp (Osram) and Chroma ET-GFP (49002) or Chroma ET-DsRed (49005) filter sets. Image analysis was performed by using MetaMorph software (Universal Imaging, Downington, PA). Cells were kept at 37°C during observation.

U2OS cells were microinjected in L-15 medium with dynein IC 70.1 (Sigma) or Myc (Covance) antibodies, diluted 1/10 from suppliers stock, or with purified CC1 fragment of p150^Glued^ (a gift of Dr. S. King, University of Missouri–Kansas City, USA) using an Eppendorf Micromanipulator 5171 coupled to a Transjector 5246, and cells were imaged as described above.

Images were prepared for publication using Adobe Photoshop. The images of fixed cells were modified by adjustments of levels and contrast. Live images were modified by adjustments of levels and contrast and applying Unsharp Mask and Gaussian Blur filters.

## Supporting Information

Figure S1 **Protein gel used for identification of bio-GFP-BICD2-CT binding partners by mass spectrometry.** To identify binding partners of BICD2, streptavidin pull-down assays were performed with extracts of HeLa cells expressing BirA alone or together with bio-GFP-BICD2 full-length, N-terminus, or C terminus. Proteins were separated on a 3%–8% Tris-acetate gel and stained with Colloidal Blue Staining Kit (Invitrogen). Mass spectrometry analysis of the proteins in the last lane is shown in Table S1 (the first lane served as a control).
(0.28 MB TIF)

Figure S2 **BICD2 associates with the NE in G2 phase in a RanBP2-dependent manner in U2OS cells.** (A) U2OS cells were transfected with a control siRNA or a mixture of RanBP2 siRNAs #1 and #2, fixed with cold methanol followed by paraformaldehyde 3 d later, and stained for endogenous BICD2, RanBP2, and cyclin B1. Note that BICD2 is strongly recruited to the NE in cyclin B1 positive cells and that this recruitment is blocked by RanBP2 depletion. (B) U2OS cells that were either treated with 10 µM nocodazole or transfected with control siRNAs or a mixture of RanBP2 siRNAs #1 and #2 were stained as described for (A), and the percentage of cyclin B1-positive cells showing BICD2 accumulation at the NE was counted. In case of RanBP2 knockdown, only the cells in which RanBP2-specific nuclear staining was reduced to background levels were included in the quantification. Error bars represent SD; ∼40–100 cells were counted in three experiments.
(0.66 MB TIF)

Figure S3 **RanBP2 and RanGAP1-positive cytoplasmic puncta are AL.** (A,B) Control HeLa cells or cells either treated for 1 h with 10 µM nocodazole were fixed with cold methanol and stained with the antibodies to NPC components NUP214, RanBP2, MAB414, and RanGAP1. (C) HeLa cells stably expressing POM121-YFP(3) were fixed with paraformaldehyde and stained with antibodies against RanBP2. Where indicated, cells were treated for 1 h with 10 µM nocodazole. Note complete co-localization of all NPC markers in the cytoplasmic puncta and the enlargement of these puncta after nocodazole treatment. (D) Transmission electron photomicrographs of HeLa cells treated with 10 µM nocodazole for 1 h immunostained for RanGAP1 before plastic embedding using immunoperoxidase. Cells were fixed with paraformaldehyde and stained with anti-RanGAP1 using an avidin-biotin-peroxidase complex procedure with diaminobenzidine as the chromogen yielding an electron dense precipitate. Staining is selectively associated with either the cytoplasmic face of NPCs in the NE, or with cytoplasmic ensembles of nuclear pore-like structures also referred to as AL (arrows). Nu, nucleus; m, mitochondrion; bars, 250 nm. This result is fully consistent with previous descriptions of AL [49] and shows that RanGAP1 is a good marker for these structures.
(3.88 MB TIF)

Figure S4 **Protein depletion in HeLa and U2OS cells.** Western blots with the indicated antibodies were performed with equal amounts of extracts of HeLa or U2OS cells 3 d after transfection with the indicated siRNAs. Note that dynein, KIF5B, BICD2, and NPC components can be depleted independently of each other. The knockdown of dynein HC also causes depletion of dynein IC, in agreement with published data [13].
(0.38 MB DOC)

Figure S5 **GFP fusions of BICD2 and BICD2 C terminus co-localize with nuclear pores on the NE.** HeLa cells were transfected with the indicated GFP fusions, treated with 10 µM nocodazole, pre-extracted in a buffer with 0.5% Triton X-100, fixed with paraformaldehyde, and stained for RanBP2. In the overlays, BICD2 is shown in green and RanBP2 in red. Note specific co-localization of the GFP fusions with the NPCs.
(0.98 MB TIF)

Figure S6 **BICD2 is required for targeting dynein/dynactin to the NE and AL.** (A) HeLa cells were treated with 10 µM nocodazole for 1 h, fixed with cold methanol, and stained for the indicated endogenous proteins. Dynactin is visualized with antibody to Arp1 and p50/dynamitin. Colors used for the overlays are indicated above the corresponding images. Note that in cells where BICD2 associates with RanBP2-positive NE and AL, dynactin subunits are also targeted to these structures. (B) HeLa cells were transfected with a control siRNA (upper panel) or BICD2#1 siRNA (bottom panel). Three days later, cells were treated with 10 µM nocodazole for 5 h, fixed with cold methanol, and stained for endogenous RanBP2, phospho-histone H3, and dynactin (p150^Glued^). NE staining by dynactin antibodies is indicated by an arrow. Colors used for the overlays are indicated above the corresponding images. Note that dynactin is enriched at the NE and annulate lamellae in control phospho-histone H3 positive cell, but not in BICD2-knockdown cell. (C) Percentage of HeLa cells positive for phospho-histone H3 that show strong accumulation of dynactin at the RanBP2-positive NE and AL in control or BICD2-depleted cells 3 d after siRNA transfection. Only the cells with clearly visible AL were included in the quantification. Error bars represent SD; ∼25–30 cells were counted in two experiments.
(2.51 MB TIF)

Figure S7 **Depletion of dynein and dynactin causes separation of centrosomes and nuclei in prophase U2OS and HeLa cells.** (A) mCherry-α-tubulin stable U2OS cell line was imaged with a 3 min time interval 2.5 d after transfection with the indicated siRNA mixtures. Note that the centrosomes separate completely from the NE envelope in a dynein-depleted cell and that this effect is rescued by co-depletion of KIF5B. 0 min indicates the first frame after NEB (defined as the time when mCherry-α-tubulin entered the nucleus). (B) mCherry-α-tubulin stable HeLa cell line was imaged with a 2 or 3 min time interval 2.5 d after transfection with the control, p150^Glued^, or DHC#2 siRNAs. 0 min indicates the first frame after NEB (defined as the time when mCherry-α-tubulin entered the nucleus). Note that the centrosomes separate completely from the NE envelope in a dynein- or dynactin-depleted cell.
(1.50 MB TIF)

Figure S8 **Microinjection of the recombinant coiled coil fragment 1 (CC1) of p150^Glued^ causes rapid separation of centrosomes and the nucleus in prophase cells.** U2OS cells stably expressing mCherry-α-tubulin were microinjected with recombinant CC1 at the needle concentration 0.85 mg/ml. Late G2 cells were chosen based on the presence of separated centrosomes. Time t = 0:00 indicates the time-point just prior to injection.
(0.29 MB TIF)

Figure S9 **Characterization of a HeLa cell line stably expressing GFP-RanGAP1.** Western blots prepared with equal amounts of extracts of control HeLa cells or the stable GFP-RanGAP1 HeLa cell line and incubated with antibodies against GFP or RanGAP1. Note that the expression levels of the fusion protein exceeded the endogenous RanGAP1 levels by approximately a factor of 4; however, the amount of SUMOylated RanGAP1, which is likely to be RanBP2- and NPC-bound [23],[24], was not significantly altered compared to control cells; in the stable cell line, this pool was predominantly represented by the GFP-tagged RanGAP1.
(0.14 MB TIF)

Figure S10 **The effect of MT motor depletion on AL distribution.** (A) HeLa cells were transfected with siRNAs against KIF5B, fixed with cold methanol 3 d later, and stained for RanBP2 and γ-tubulin. Note that AL accumulate as a single pericentrosomal dot in a cell with a single centrosome and around both centrosomes after their separation. (B) HeLa cells were transfected with KIF5B or DHC siRNAs, fixed with methanol, and stained for endogenous NUP214, RanBP2, and with the MAB414 antibody. (C) HeLa cells stably expressing POM121-YFP(3) were transfected with KIF5B or DHC siRNAs, fixed with paraformaldehyde, and stained with antibodies against RanBP2. Note that all NPC markers show characteristic re-localization to the centrosome or to the cell periphery, supporting the view that these structures are indeed AL.
(1.65 MB TIF)

Figure S11 **Behavior of AL in BICD2-depleted cells.** (A) HeLa cells were transfected with the indicated siRNAs, fixed 3 d later with cold methanol followed by paraformaldehyde, and stained for RanBP2 and cyclin B1. Note that AL are dispersed in BICD2-depleted cyclin B1-positive cells. (B) Total area occupied by AL was measured in ∼25 cyclin B1-positive cells stained as described for (A). Error bars represent SEM. The area is decreased in KIF5B-depleted G2 cells because AL are strongly concentrated in the pericentrosomal region and enlarged in BICD2-depleted cells because AL are dispersed. (C) GFP-RanGAP1 stable HeLa cell line was imaged with a 3 min time interval 2 d after transfection with the BICD2#1 siRNA alone or in combination with siRNAs against DHC#1 or KIF5B#1. 0 min indicates the first frame after NEB. Contrast is inverted. Note that AL remain dispersed and accumulated neither at the cell periphery nor the cell center, even when dynein or KIF5B were co-depleted.
(0.93 MB TIF)

Figure S12 **BICD2 depletion blocks re-localization of AL caused by the knockdown of dynein or kinesin-1.** HeLa cells were transfected with the KIF5B#1 or DHC#1 siRNAs in combination with the control or BICD2#1 siRNAs, fixed with paraformaldehyde 3 d later, and stained for BICD2, RanBP2 (green in overlay), and cyclin B1 (red in overlay). Insets show enlargement of cyclin B1-positive dynein-depleted cells. Accumulations of AL at the two separated centrosomes in kinesin-1-depleted cells and at the cell periphery in dynein-depleted cells are indicated by arrows. Note that cells showing strong AL displacement to the centrosome or the cell periphery cannot be found in BICD2-codepleted cells.
(2.64 MB TIF)

Figure S13 **Keratin and actin are not directly involved in G2-specific displacement of nuclei and AL after dynein and kinesin-1 depletion.** (A) HeLa cells were transfected with the indicated siRNAs, fixed with cold methanol 3 d later, and stained for keratin, α-tubulin, and RanGAP1. Note that keratin network looks similar in dynein HC and KIF5B-delpeted G2 cells (which are identified by the characteristic position of the AL). This is in agreement with published data indicating that keratin distribution is not significantly affected by microtubule motors [67]. (B) HeLa cells were transfected with the indicated siRNAs; 2 d later, cells were incubated overnight with 1 µm cytochalasin D or left untreated. At 3 d post-transfection, cells were fixed with paraformaldehyde and stained with antibodies to α-tubulin and RanGAP1, and with fluorescent phalloidin, to visualize the actin network. Note that cytochalasin-induced disassembly of actin fibers does not prevent separation of centrosomes (arrows) and nuclei in a dynein-depleted cell or AL aggregation in KIF5B-depleted cell.
(1.92 MB TIF)

Figure S14 **Vimentin distribution is affected by knockdown of dynein and kinesin-1 but does not correlate with G2-specific displacement of nuclei and AL.** (A,B) HeLa cells were transfected with the indicated siRNAs, fixed with cold methanol 3 d later, and stained for vimentin and RanGAP1. A cell with the characteristic G2-specific aggregation of AL caused by KIF5B depletion is indicated by an arrow. (B) shows a cell with the characteristic G2-specific peripheral displacement of AL and the nucleus caused by dynein HC depletion; note that vimentin network is not re-localized to the cell corner together with the nucleus. (C) Average fluorescence intensity of vimentin staining in the perinuclear region normalized to the average fluorescence intensity of the whole cell (expressed in %). Intensity of a circular area (3 µm in diameter) was measured in ∼20 cells; background was subtracted. In case of dynein HC and KIF5B knockdowns, two cell populations were analyzed: cells with randomly dispersed AL (corresponding to G1 and S phase) and cells with strongly aggregated (KIF5B depletion) or peripherally located (dynein HC depletion) AL, which are in G2 phase. For the other conditions, cell cycle stages were not discriminated. In agreement with published data, the distribution of vimentin became more concentrated in the center of the cell after KIF5B knockdown and shifted to the cell periphery after dynein knockdown (A,C) [67]. However, in contrast to the positioning of the nuclei and AL, the distribution of vimentin was cell-cycle independent (C). Moreover, in dynein-depleted cells that showed a strong displacement of the nuclei into one cell corner, vimentin network remained in the central part of the cell, indicating that vimentin redistribution is not the underlying cause of the nuclear movement (B).
(1.73 MB TIF)

Table S1 **Identification of BICD2-CT binding partners by mass spectrometry in HeLa cell extract.** The table shows the proteins identified with a significant Mascot score in the pull-down with streptavidin beads from an extract of HeLa cells co-expressing Bio-GFP-BICD2-CT (BICD2 amino acids 487–820) and biotin ligase BirA. A pull-down from HeLa cells expressing BirA alone was used as a control (only proteins that displayed significantly higher Mascot score in the Bio-GFP-BICD2-CT lane compared to the control lane are listed). The proteins were separated on 3%–8% polyacrylamide gel (Figure S1); proteins smaller than 50 kDa were not analyzed in this experiment. For each identified protein, the list is filtered for duplicates and shows only the hits with the highest score and most identified peptides.
(0.03 MB DOC)

Video S1 **Centrosome separation in control prophase cells.** Control HeLa cells expressing mCherry-α-tubulin were imaged with a 3 min interval for 5 h and 30 min.
(2.74 MB MOV)

Video S2 **Centrosome separation in dynactin (p150^Glued^)-depleted prophase cells.** HeLa cells expressing mCherry-α-tubulin were transfected with the siRNA against p150^Glued^ and imaged 2 d later with a 3 min interval for 5 h and 30 min.
(0.44 MB MOV)

Video S3 **Centrosome separation in dynein-depleted prophase cells.** HeLa cells expressing mCherry-α-tubulin were transfected with the DHC#2 siRNA and imaged 2 d later with a 2 min interval for 4 h and 20 min.
(1.19 MB MPG)

Video S4 **Centrosome separation in control prophase cells.** Control U2OS cells expressing mCherry-α-tubulin were imaged with a 5 min interval for 1 h and 50 min.
(0.73 MB MOV)

Video S5 **Centrosome separation in dynein-depleted prophase cells.** U2OS cells expressing mCherry-α-tubulin were transfected with the DHC#1 siRNA and imaged 2.5 d later with a 3 min interval for 2 h and 30 min.
(0.69 MB MOV)

Video S6 **Peripheral displacement of the nucleus in dynein-depleted cells prior to mitotic entry.** HeLa cells stably expressing GFP-RanGAP1 were transfected with the DHC#2 siRNA and imaged 2 d later with a 3 min interval for 8 h and 30 min.
(1.17 MB MOV)

Video S7 **Peripheral displacement of the nuclei in dynein-depleted cells prior to mitotic entry.** HeLa cells stably expressing GFP-RanGAP1 were transfected with the DHC#2 siRNA and imaged 2 d later with a 3 min interval for 5 h and 30 min. Note abrupt separation of the nuclei.
(0.42 MB MOV)

Video S8 **Behavior of the AL in control cells.** HeLa cells stably expressing GFP-RanGAP1 were imaged with a 3 min interval for 11 h (every second frame is shown).
(1.52 MB MOV)

Video S9 **Behavior of the AL in dynactin (p150^Glued^)-depleted cells.** HeLa cells stably expressing GFP-RanGAP1 were transfected with the siRNA against p150^Glued^ and imaged 2 d later with a 2 min interval for 6 h.
(4.43 MB MOV)

Video S10 **Behavior of the AL in KIF5B-depleted cells.** HeLa cells stably expressing GFP-RanGAP1 were transfected with the KIF5B#1 siRNA and imaged 2 d later with a 3 min interval for 6 h and 30 min.
(1.57 MB MOV)

Video S11 **Behavior of the AL in BICD2-depleted cells.** HeLa cells stably expressing GFP-RanGAP1 were transfected with the BICD2#1 siRNA and imaged with a 3 min interval for 5 h and 24 min.
(4.24 MB MOV)

Video S12 **Behavior of the AL in dynein/BICD2-depleted cells.** HeLa cells stably expressing GFP-RanGAP1 were co-transfected with the DHC#2 and BICD2#1 siRNAs and imaged 2 d later with a 3 min interval for 6 h and 6 min.
(1.45 MB MOV)

Video S13 **Behavior of the AL in KIF5B/BICD2-depleted cells.** HeLa cells stably expressing GFP-RanGAP1 were co-transfected with the KIF5B#1and BICD2#1 siRNAs and imaged 2 d later with a 3 min interval for 8 h.
(3.15 MB MOV)

## Abbreviations

AL: annulate lamellae
BICD: Bicaudal D
co-IP: co-immunoprecipitation
GST: glutathione S-transferase
HC: heavy chain
IC: intermediate chain
MT: microtubule
NE: nuclear envelope
NEB: NE breakdown
NPC: nuclear pore complex
STLC: S-trityl-L-cysteine

## References

[1] Vale R. D (2003). The molecular motor toolbox for intracellular transport.. Cell.

[2] Karki S, Holzbaur E. L (1999). Cytoplasmic dynein and dynactin in cell division and intracellular transport.. Curr Opin Cell Biol.

[3] Gross S. P (2004). Hither and yon: a review of bi-directional microtubule-based transport.. Phys Biol.

[4] Welte M. A (2004). Bidirectional transport along microtubules.. Curr Biol.

[5] Jordens I, Marsman M, Kuijl C, Neefjes J (2005). Rab proteins, connecting transport and vesicle fusion.. Traffic.

[6] Karcher R. L, Deacon S. W, Gelfand V. I (2002). Motor-cargo interactions: the key to transport specificity.. Trends Cell Biol.

[7] Claussen M, Suter B (2005). BicD-dependent localization processes: from Drosophilia development to human cell biology.. Ann Anat.

[8] Hoogenraad C. C, Akhmanova A, Howell S. A, Dortland B. R, De Zeeuw C. I (2001). Mammalian Golgi-associated Bicaudal-D2 functions in the dynein-dynactin pathway by interacting with these complexes.. Embo J.

[9] Hoogenraad C. C, Wulf P, Schiefermeier N, Stepanova T, Galjart N (2003). Bicaudal D induces selective dynein-mediated microtubule minus end-directed transport.. Embo J.

[10] Short B, Preisinger C, Schaletzky J, Kopajtich R, Barr F. A (2002). The Rab6 GTPase regulates recruitment of the dynactin complex to Golgi membranes.. Curr Biol.

[11] Matanis T, Akhmanova A, Wulf P, Del Nery E, Weide T (2002). Bicaudal-D regulates COPI-independent Golgi-ER transport by recruiting the dynein-dynactin motor complex.. Nat Cell Biol.

[12] Januschke J, Nicolas E, Compagnon J, Formstecher E, Goud B (2007). Rab6 and the secretory pathway affect oocyte polarity in Drosophila.. Development.

[13] Grigoriev I, Splinter D, Keijzer N, Wulf P. S, Demmers J (2007). Rab6 regulates transport and targeting of exocytotic carriers.. Dev Cell.

[14] Bullock S. L, Ish-Horowicz D (2001). Conserved signals and machinery for RNA transport in Drosophila oogenesis and embryogenesis.. Nature.

[15] Bullock S. L, Nicol A, Gross S. P, Zicha D (2006). Guidance of bidirectional motor complexes by mRNA cargoes through control of dynein number and activity.. Curr Biol.

[16] Mach J. M, Lehmann R (1997). An Egalitarian-BicaudalD complex is essential for oocyte specification and axis determination in Drosophila.. Genes Dev.

[17] Dienstbier M, Boehl F, Li X, Bullock S. L (2009). Egalitarian is a selective RNA-binding protein linking mRNA localization signals to the dynein motor.. Genes Dev.

[18] Larsen K. S, Xu J, Cermelli S, Shu Z, Gross S. P (2008). BicaudalD actively regulates microtubule motor activity in lipid droplet transport.. PLoS ONE.

[19] Wu J, Matunis M. J, Kraemer D, Blobel G, Coutavas E (1995). Nup358, a cytoplasmically exposed nucleoporin with peptide repeats, Ran-GTP binding sites, zinc fingers, a cyclophilin A homologous domain, and a leucine-rich region.. J Biol Chem.

[20] Yokoyama N, Hayashi N, Seki T, Pante N, Ohba T (1995). A giant nucleopore protein that binds Ran/TC4.. Nature.

[21] Gorlich D, Kutay U (1999). Transport between the cell nucleus and the cytoplasm.. Annu Rev Cell Dev Biol.

[22] Pichler A, Gast A, Seeler J. S, Dejean A, Melchior F (2002). The nucleoporin RanBP2 has SUMO1 E3 ligase activity.. Cell.

[23] Matunis M. J, Coutavas E, Blobel G (1996). A novel ubiquitin-like modification modulates the partitioning of the Ran-GTPase-activating protein RanGAP1 between the cytosol and the nuclear pore complex.. J Cell Biol.

[24] Mahajan R, Delphin C, Guan T, Gerace L, Melchior F (1997). A small ubiquitin-related polypeptide involved in targeting RanGAP1 to nuclear pore complex protein RanBP2.. Cell.

[25] Busson S, Dujardin D, Moreau A, Dompierre J, De Mey J. R (1998). Dynein and dynactin are localized to astral microtubules and at cortical sites in mitotic epithelial cells.. Curr Biol.

[26] Salina D, Bodoor K, Eckley D. M, Schroer T. A, Rattner J. B (2002). Cytoplasmic dynein as a facilitator of nuclear envelope breakdown.. Cell.

[27] Hetzer M. W, Walther T. C, Mattaj I. W (2005). Pushing the envelope: structure, function, and dynamics of the nuclear periphery.. Annu Rev Cell Dev Biol.

[28] Rosenblatt J (2005). Spindle assembly: asters part their separate ways.. Nat Cell Biol.

[29] Robinson J. T, Wojcik E. J, Sanders M. A, McGrail M, Hays T. S (1999). Cytoplasmic dynein is required for the nuclear attachment and migration of centrosomes during mitosis in Drosophila.. J Cell Biol.

[30] Gonczy P, Pichler S, Kirkham M, Hyman A. A (1999). Cytoplasmic dynein is required for distinct aspects of MTOC positioning, including centrosome separation, in the one cell stage Caenorhabditis elegans embryo.. J Cell Biol.

[31] Stelter P, Kunze R, Flemming D, Hopfner D, Diepholz M (2007). Molecular basis for the functional interaction of dynein light chain with the nuclear-pore complex.. Nat Cell Biol.

[32] Malone C. J, Misner L, Le Bot N, Tsai M. C, Campbell J. M (2003). The C. elegans hook protein, ZYG-12, mediates the essential attachment between the centrosome and nucleus.. Cell.

[33] Zhang X, Lei K, Yuan X, Wu X, Zhuang Y (2009). SUN1/2 and Syne/Nesprin-1/2 complexes connect centrosome to the nucleus during neurogenesis and neuronal migration in mice.. Neuron.

[34] de Boer E, Rodriguez P, Bonte E, Krijgsveld J, Katsantoni E (2003). Efficient biotinylation and single-step purification of tagged transcription factors in mammalian cells and transgenic mice.. Proc Natl Acad Sci U S A.

[35] Lansbergen G, Komarova Y, Modesti M, Wyman C, Hoogenraad C. C (2004). Conformational changes in CLIP-170 regulate its binding to microtubules and dynactin localisation.. J Cell Biol.

[36] Cai Y, Singh B. B, Aslanukov A, Zhao H, Ferreira P. A (2001). The docking of kinesins, KIF5B and KIF5C, to Ran-binding protein 2 (RanBP2) is mediated via a novel RanBP2 domain.. J Biol Chem.

[37] Cho K. I, Cai Y, Yi H, Yeh A, Aslanukov A (2007). Association of the kinesin-binding domain of RanBP2 to KIF5B and KIF5C determines mitochondria localization and function.. Traffic.

[38] Cho K. I, Yi H, Desai R, Hand A. R, Haas A. L (2009). RANBP2 is an allosteric activator of the conventional kinesin-1 motor protein, KIF5B, in a minimal cell-free system.. EMBO Rep.

[39] Kessel R. G (1992). Annulate lamellae: a last frontier in cellular organelles.. Int Rev Cytol.

[40] Davis L. I, Blobel G (1986). Identification and characterization of a nuclear pore complex protein.. Cell.

[41] Hutten S, Kehlenbach R. H (2006). Nup214 is required for CRM1-dependent nuclear protein export in vivo.. Mol Cell Biol.

[42] Imreh G, Hallberg E (2000). An integral membrane protein from the nuclear pore complex is also present in the annulate lamellae: implications for annulate lamella formation.. Exp Cell Res.

[43] Hsu J. Y, Sun Z. W, Li X, Reuben M, Tatchell K (2000). Mitotic phosphorylation of histone H3 is governed by Ipl1/aurora kinase and Glc7/PP1 phosphatase in budding yeast and nematodes.. Cell.

[44] Dujardin D. L, Vallee R. B (2002). Dynein at the cortex.. Curr Opin Cell Biol.

[45] Tanenbaum M. E, Macurek L, Galjart N, Medema R. H (2008). Dynein, Lis1 and CLIP-170 counteract Eg5-dependent centrosome separation during bipolar spindle assembly.. Embo J.

[46] Quintyne N. J, Gill S. R, Eckley D. M, Crego C. L, Compton D. A (1999). Dynactin is required for microtubule anchoring at centrosomes.. J Cell Biol.

[47] Ketema M, Wilhelmsen K, Kuikman I, Janssen H, Hodzic D (2007). Requirements for the localization of nesprin-3 at the nuclear envelope and its interaction with plectin.. J Cell Sci.

[48] Kapitein L. C, Peterman E. J, Kwok B. H, Kim J. H, Kapoor T. M (2005). The bipolar mitotic kinesin Eg5 moves on both microtubules that it crosslinks.. Nature.

[49] Cordes V. C, Reidenbach S, Franke W. W (1996). Cytoplasmic annulate lamellae in cultured cells: composition, distribution, and mitotic behavior.. Cell Tissue Res.

[50] Hebbar S, Mesngon M. T, Guillotte A. M, Desai B, Ayala R (2008). Lis1 and Ndel1 influence the timing of nuclear envelope breakdown in neural stem cells.. J Cell Biol.

[51] Wilhelmsen K, Ketema M, Truong H, Sonnenberg A (2006). KASH-domain proteins in nuclear migration, anchorage and other processes.. J Cell Sci.

[52] Schneider M, Noegel A. A, Karakesisoglou I (2008). KASH-domain proteins and the cytoskeletal landscapes of the nuclear envelope.. Biochem Soc Trans.

[53] Roux K. J, Crisp M. L, Liu Q, Kim D, Kozlov S (2009). Nesprin 4 is an outer nuclear membrane protein that can induce kinesin-mediated cell polarization.. Proc Natl Acad Sci U S A.

[54] Liu Q, Pante N, Misteli T, Elsagga M, Crisp M (2007). Functional association of Sun1 with nuclear pore complexes.. J Cell Biol.

[55] Cimini D (2008). Merotelic kinetochore orientation, aneuploidy, and cancer.. Biochim Biophys Acta.

[56] Beaudouin J, Gerlich D, Daigle N, Eils R, Ellenberg J (2002). Nuclear envelope breakdown proceeds by microtubule-induced tearing of the lamina.. Cell.

[57] Tsai L. H, Gleeson J. G (2005). Nucleokinesis in neuronal migration.. Neuron.

[58] Driegen S, Ferreira R, van Zon A, Strouboulis J, Jaegle M (2005). A generic tool for biotinylation of tagged proteins in transgenic mice.. Transgenic Res.

[59] Shaner N. C, Campbell R. E, Steinbach P. A, Giepmans B. N, Palmer A. E (2004). Improved monomeric red, orange and yellow fluorescent proteins derived from Discosoma sp. red fluorescent protein.. Nat Biotechnol.

[60] Wilhelmsen K, Litjens S. H, Kuikman I, Tshimbalanga N, Janssen H (2005). Nesprin-3, a novel outer nuclear membrane protein, associates with the cytoskeletal linker protein plectin.. J Cell Biol.

[61] Joseph J, Liu S. T, Jablonski S. A, Yen T. J, Dasso M (2004). The RanGAP1-RanBP2 complex is essential for microtubule-kinetochore interactions in vivo.. Curr Biol.

[62] Grigoriev I, Gouveia S. M, van der Vaart B, Demmers J, Smyth J. T (2008). STIM1 is a MT-plus-end-tracking protein involved in remodeling of the ER.. Curr Biol.

[63] Lansbergen G, Grigoriev I, Mimori-Kiyosue Y, Ohtsuka T, Higa S (2006). CLASPs attach microtubule plus ends to the cell cortex through a complex with LL5beta.. Dev Cell.

[64] Mimori-Kiyosue Y, Grigoriev I, Lansbergen G, Sasaki H, Matsui C (2005). CLASP1 and CLASP2 bind to EB1 and regulate microtubule plus-end dynamics at the cell cortex.. J Cell Biol.

[65] Tanenbaum M. E, Galjart N, van Vugt M. A, Medema R. H (2006). CLIP-170 facilitates the formation of kinetochore-microtubule attachments.. Embo J.

[66] Hutten S, Flotho A, Melchior F, Kehlenbach R. H (2008). The Nup358-RanGAP complex is required for efficient importin {alpha}/{beta}-dependent nuclear import.. Mol Biol Cell.

[67] Helfand B. T, Chang L, Goldman R. D (2004). Intermediate filaments are dynamic and motile elements of cellular architecture.. J Cell Sci.

