# Supplementary figures and images for: Bicaudal D2, Dynein, and Kinesin-1 Associate with Nuclear Pore Complexes and Regulate Centrosome and Nuclear Positioning during Mitotic Entry
Source: PLoS Biol. 2010 Apr 6;8(4):e1000350. doi: 10.1371/journal.pbio.1000350 (PMC2850381; doi:10.1371/journal.pbio.1000350)

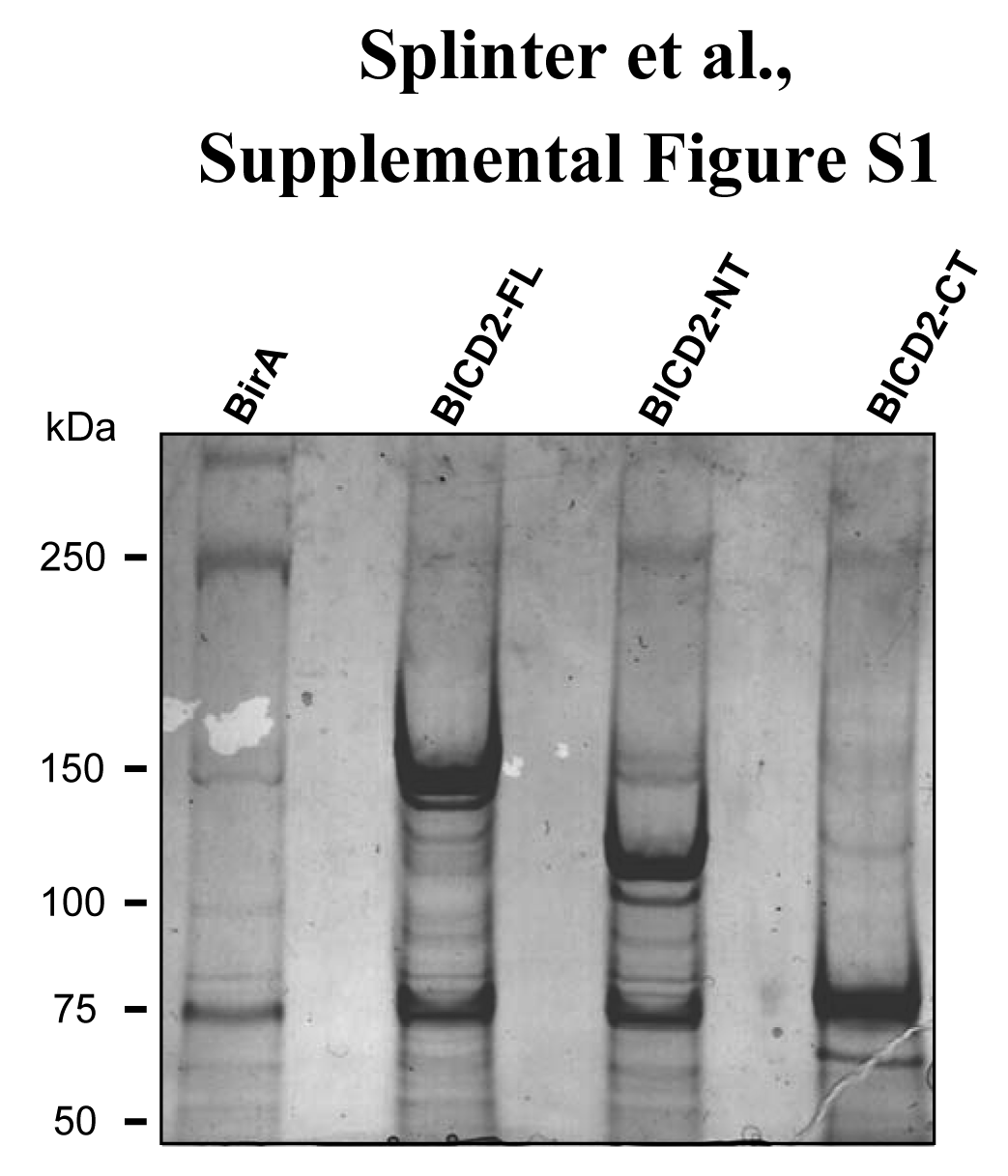

Supplement: Figure S1 — Protein gel used for identification of bio-GFP-BICD2-CT binding partners by mass spectrometry. To identify binding partners of BICD2, streptavidin pull-down assays were performed with extracts of HeLa cells expressing BirA alone or together with bio-GFP-BICD2 full-length, N-terminus, or C terminus. Proteins were separated on a 3%–8% Tris-acetate gel and stained with Colloidal Blue Staining Kit (Invitrogen). Mass spectrometry analysis of the proteins in the last lane is shown in Table S1 (the first lane served as a control). (0.28 MB TIF) [file pbio.1000350.s001.tif]

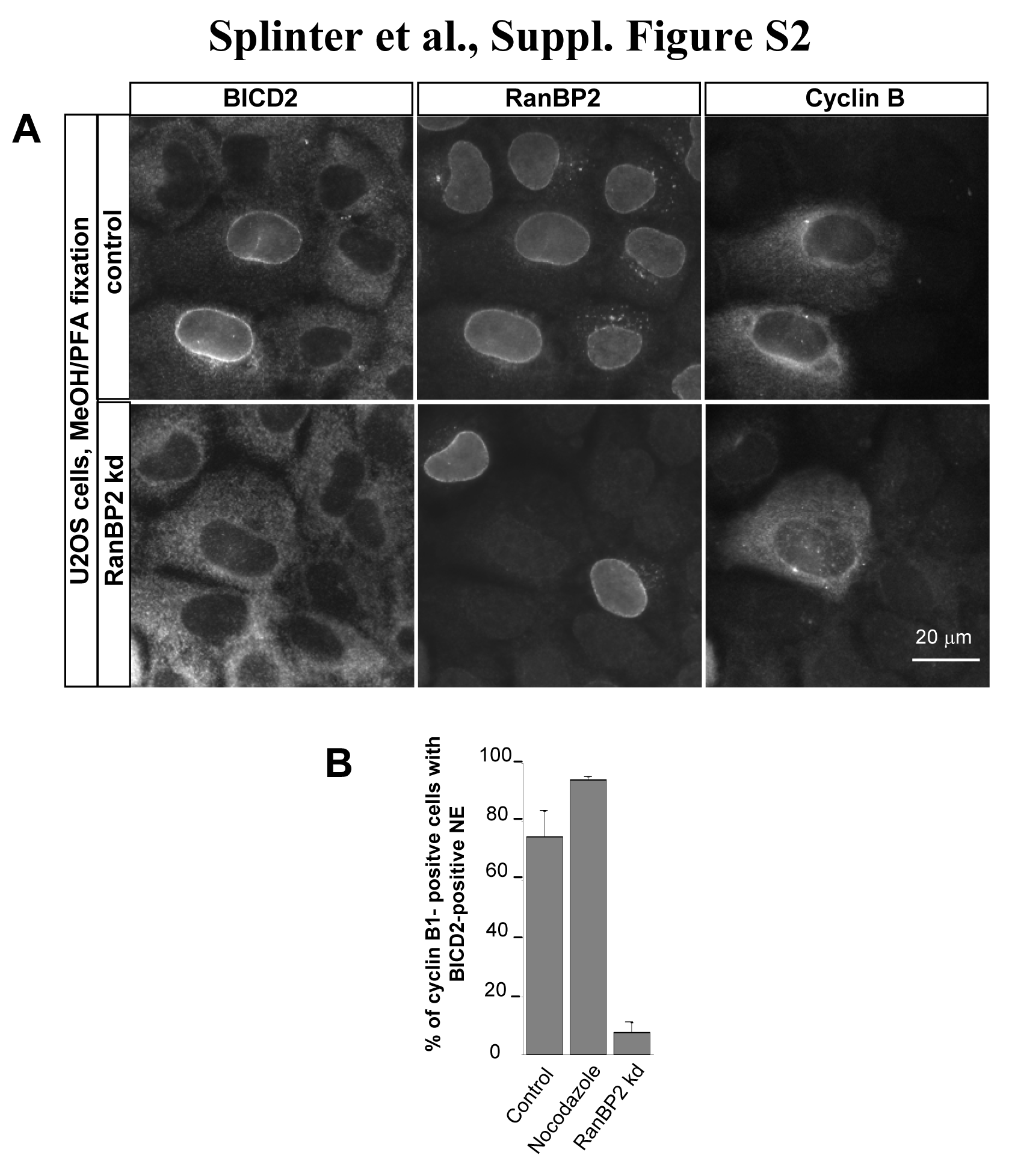

Supplement: Figure S2 — BICD2 associates with the NE in G2 phase in a RanBP2-dependent manner in U2OS cells. (A) U2OS cells were transfected with a control siRNA or a mixture of RanBP2 siRNAs #1 and #2, fixed with cold methanol followed by paraformaldehyde 3 d later, and stained for endogenous BICD2, RanBP2, and cyclin B1. Note that BICD2 is strongly recruited to the NE in cyclin B1 positive cells and that this recruitment is blocked by RanBP2 depletion. (B) U2OS cells that were either treated with 10 µM nocodazole or transfected with control siRNAs or a mixture of RanBP2 siRNAs #1 and #2 were stained as described for (A), and the percentage of cyclin B1-positive cells showing BICD2 accumulation at the NE was counted. In case of RanBP2 knockdown, only the cells in which RanBP2-specific nuclear staining was reduced to background levels were included in the quantification. Error bars represent SD; ∼40–100 cells were counted in three experiments. (0.66 MB TIF) [file pbio.1000350.s002.tif]

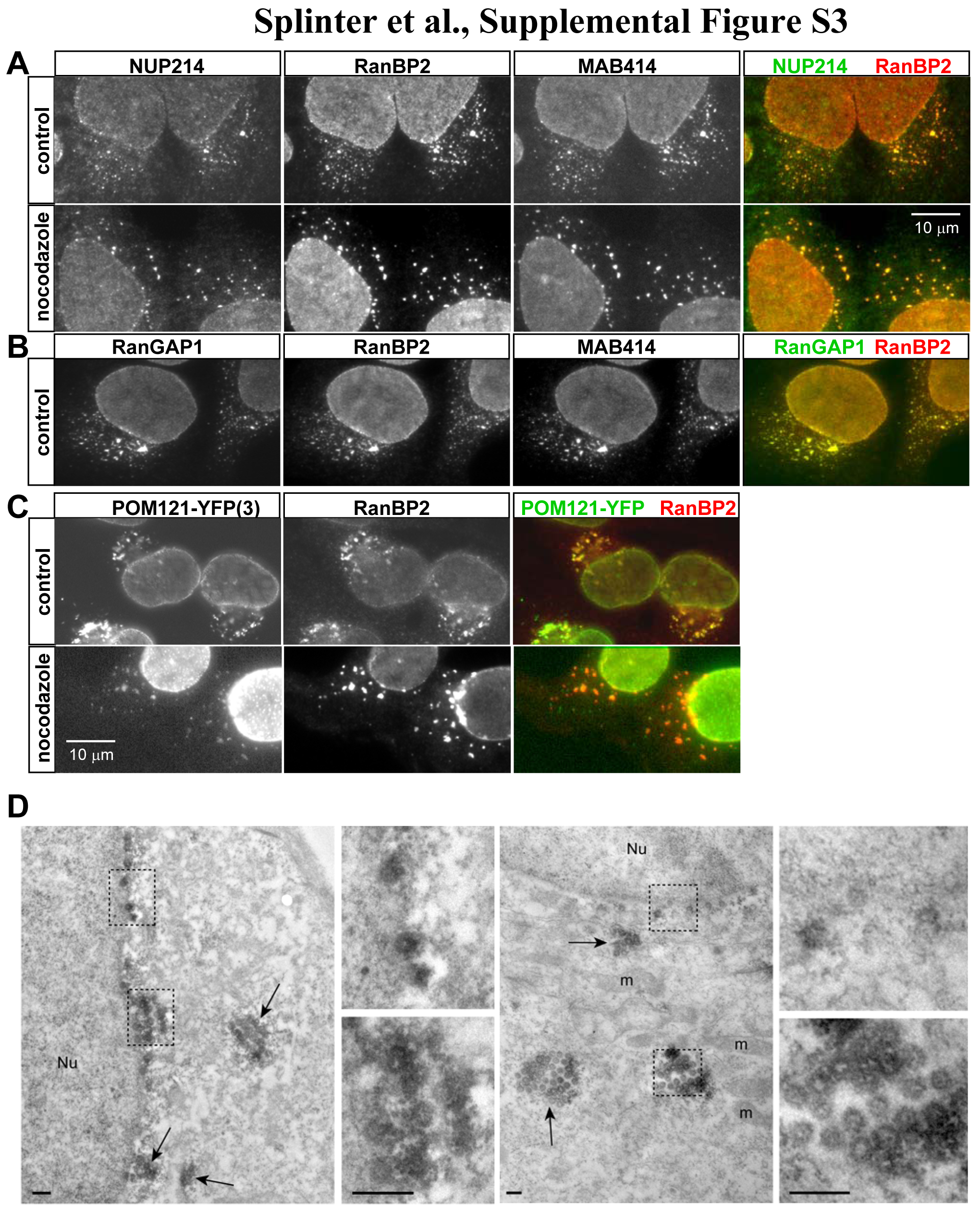

Supplement: Figure S3 — RanBP2 and RanGAP1-positive cytoplasmic puncta are AL. (A,B) Control HeLa cells or cells either treated for 1 h with 10 µM nocodazole were fixed with cold methanol and stained with the antibodies to NPC components NUP214, RanBP2, MAB414, and RanGAP1. (C) HeLa cells stably expressing POM121-YFP(3) were fixed with paraformaldehyde and stained with antibodies against RanBP2. Where indicated, cells were treated for 1 h with 10 µM nocodazole. Note complete co-localization of all NPC markers in the cytoplasmic puncta and the enlargement of these puncta after nocodazole treatment. (D) Transmission electron photomicrographs of HeLa cells treated with 10 µM nocodazole for 1 h immunostained for RanGAP1 before plastic embedding using immunoperoxidase. Cells were fixed with paraformaldehyde and stained with anti-RanGAP1 using an avidin-biotin-peroxidase complex procedure with diaminobenzidine as the chromogen yielding an electron dense precipitate. Staining is selectively associated with either the cytoplasmic face of NPCs in the NE, or with cytoplasmic ensembles of nuclear pore-like structures also referred to as AL (arrows). Nu, nucleus; m, mitochondrion; bars, 250 nm. This result is fully consistent with previous descriptions of AL [49] and shows that RanGAP1 is a good marker for these structures. (3.88 MB TIF) [file pbio.1000350.s003.tif]

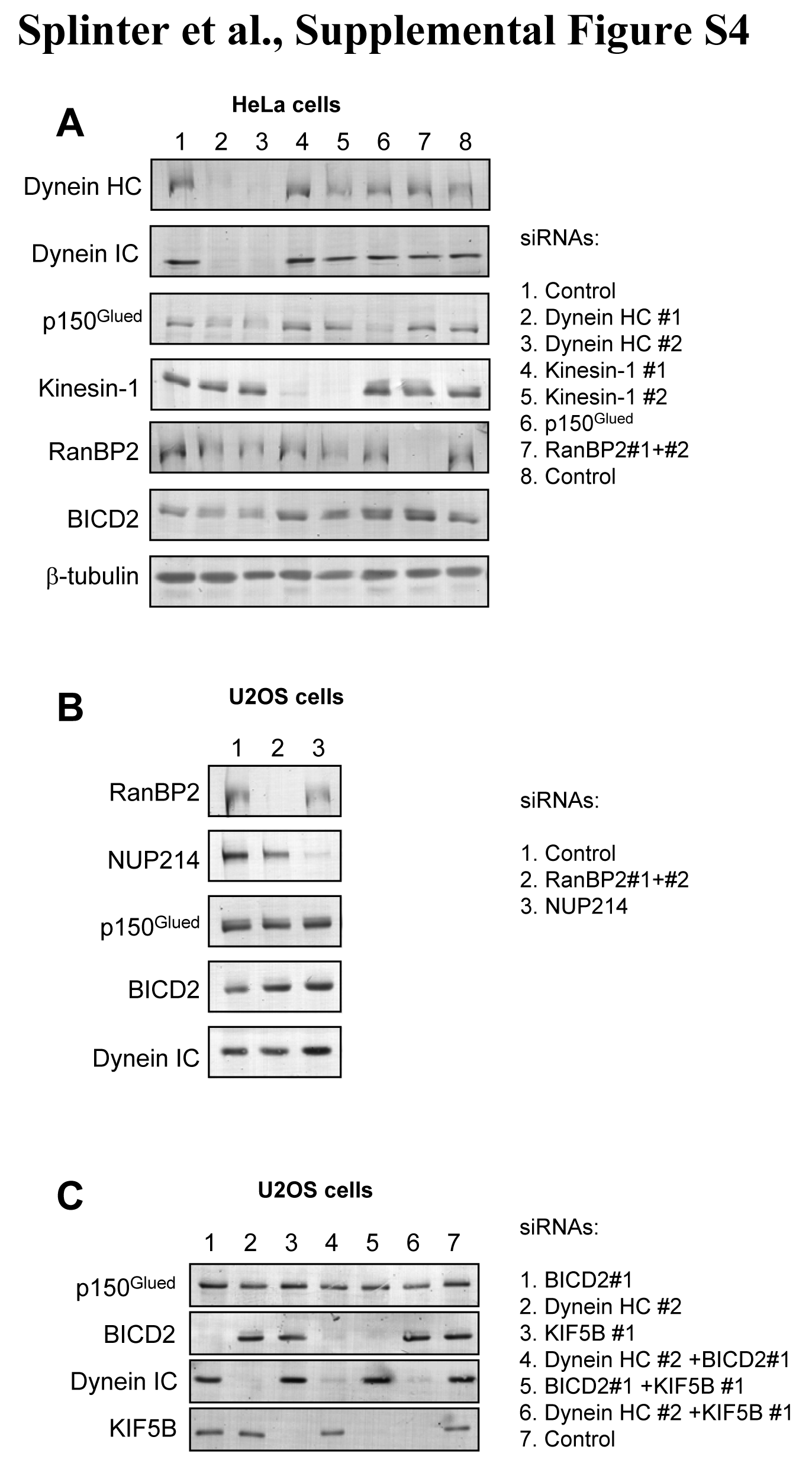

Supplement: Figure S4 — Protein depletion in HeLa and U2OS cells. Western blots with the indicated antibodies were performed with equal amounts of extracts of HeLa or U2OS cells 3 d after transfection with the indicated siRNAs. Note that dynein, KIF5B, BICD2, and NPC components can be depleted independently of each other. The knockdown of dynein HC also causes depletion of dynein IC, in agreement with published data [13]. (0.38 MB DOC) [file pbio.1000350.s004.tif]

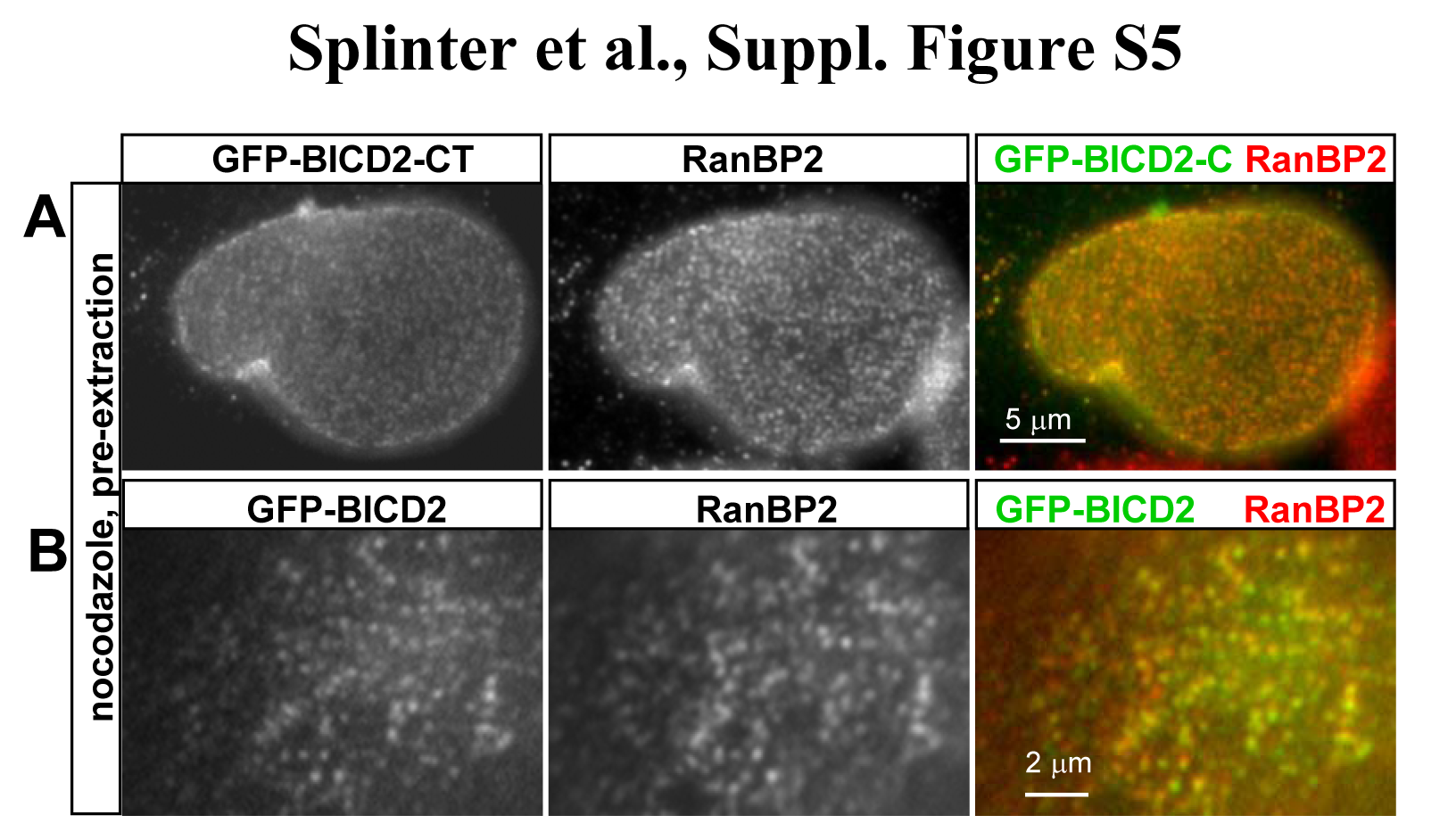

Supplement: Figure S5 — GFP fusions of BICD2 and BICD2 C terminus co-localize with nuclear pores on the NE. HeLa cells were transfected with the indicated GFP fusions, treated with 10 µM nocodazole, pre-extracted in a buffer with 0.5% Triton X-100, fixed with paraformaldehyde, and stained for RanBP2. In the overlays, BICD2 is shown in green and RanBP2 in red. Note specific co-localization of the GFP fusions with the NPCs. (0.98 MB TIF) [file pbio.1000350.s005.tif]

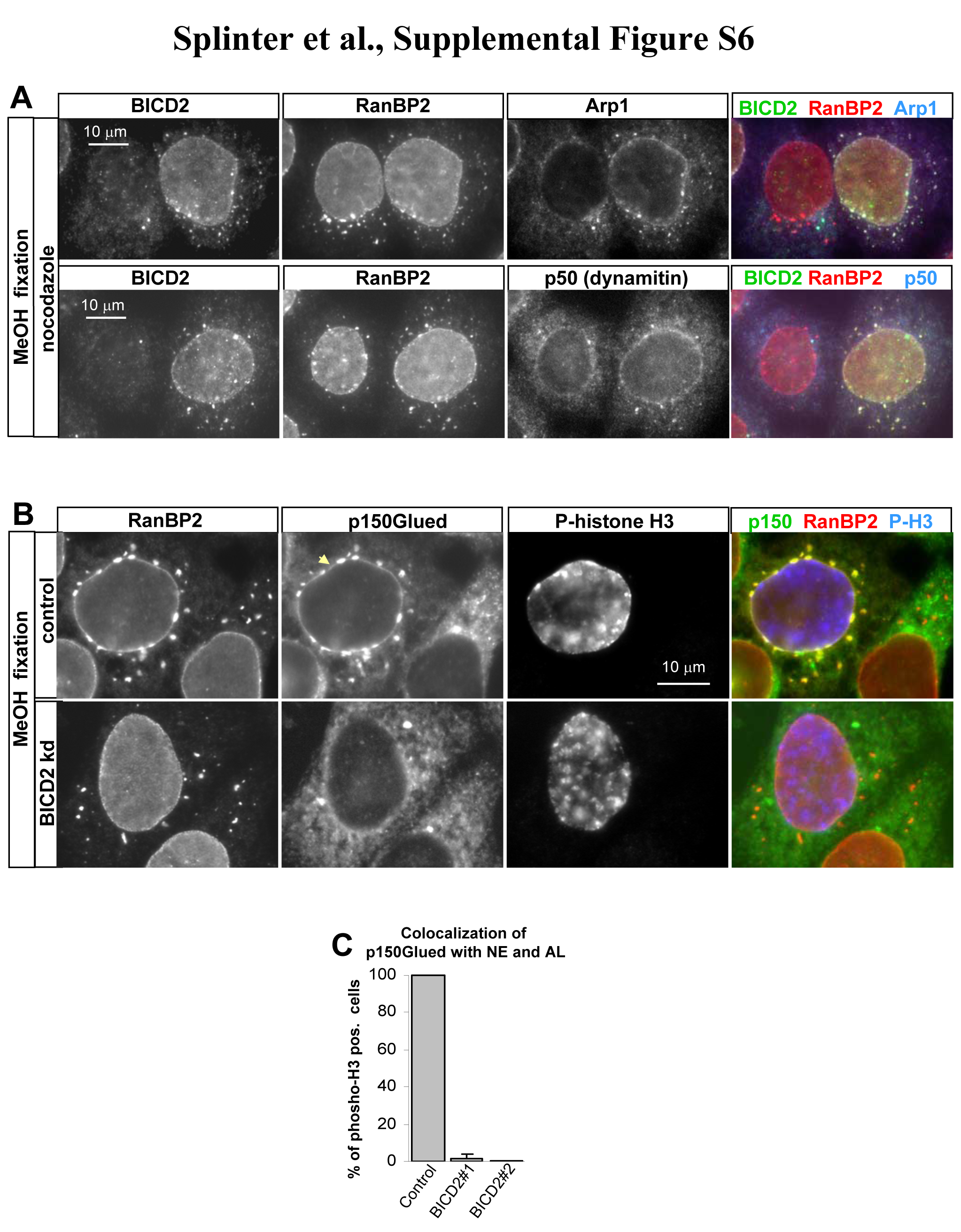

Supplement: Figure S6 — BICD2 is required for targeting dynein/dynactin to the NE and AL. (A) HeLa cells were treated with 10 µM nocodazole for 1 h, fixed with cold methanol, and stained for the indicated endogenous proteins. Dynactin is visualized with antibody to Arp1 and p50/dynamitin. Colors used for the overlays are indicated above the corresponding images. Note that in cells where BICD2 associates with RanBP2-positive NE and AL, dynactin subunits are also targeted to these structures. (B) HeLa cells were transfected with a control siRNA (upper panel) or BICD2#1 siRNA (bottom panel). Three days later, cells were treated with 10 µM nocodazole for 5 h, fixed with cold methanol, and stained for endogenous RanBP2, phospho-histone H3, and dynactin (p150Glued). NE staining by dynactin antibodies is indicated by an arrow. Colors used for the overlays are indicated above the corresponding images. Note that dynactin is enriched at the NE and annulate lamellae in control phospho-histone H3 positive cell, but not in BICD2-knockdown cell. (C) Percentage of HeLa cells positive for phospho-histone H3 that show strong accumulation of dynactin at the RanBP2-positive NE and AL in control or BICD2-depleted cells 3 d after siRNA transfection. Only the cells with clearly visible AL were included in the quantification. Error bars represent SD; ∼25–30 cells were counted in two experiments. (2.51 MB TIF) [file pbio.1000350.s006.tif]

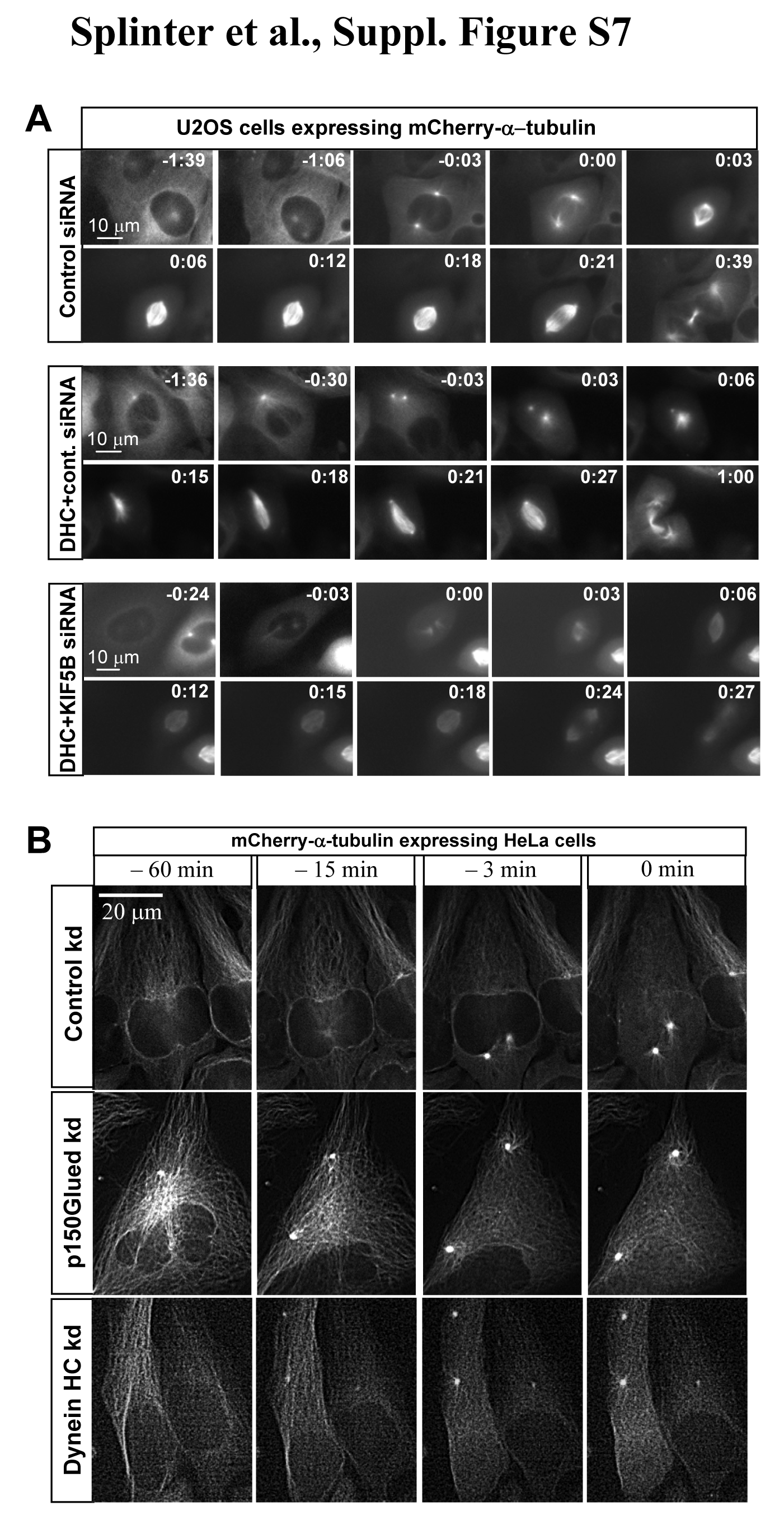

Supplement: Figure S7 — Depletion of dynein and dynactin causes separation of centrosomes and nuclei in prophase U2OS and HeLa cells. (A) mCherry-α-tubulin stable U2OS cell line was imaged with a 3 min time interval 2.5 d after transfection with the indicated siRNA mixtures. Note that the centrosomes separate completely from the NE envelope in a dynein-depleted cell and that this effect is rescued by co-depletion of KIF5B. 0 min indicates the first frame after NEB (defined as the time when mCherry-α-tubulin entered the nucleus). (B) mCherry-α-tubulin stable HeLa cell line was imaged with a 2 or 3 min time interval 2.5 d after transfection with the control, p150Glued, or DHC#2 siRNAs. 0 min indicates the first frame after NEB (defined as the time when mCherry-α-tubulin entered the nucleus). Note that the centrosomes separate completely from the NE envelope in a dynein- or dynactin-depleted cell. (1.50 MB TIF) [file pbio.1000350.s007.tif]

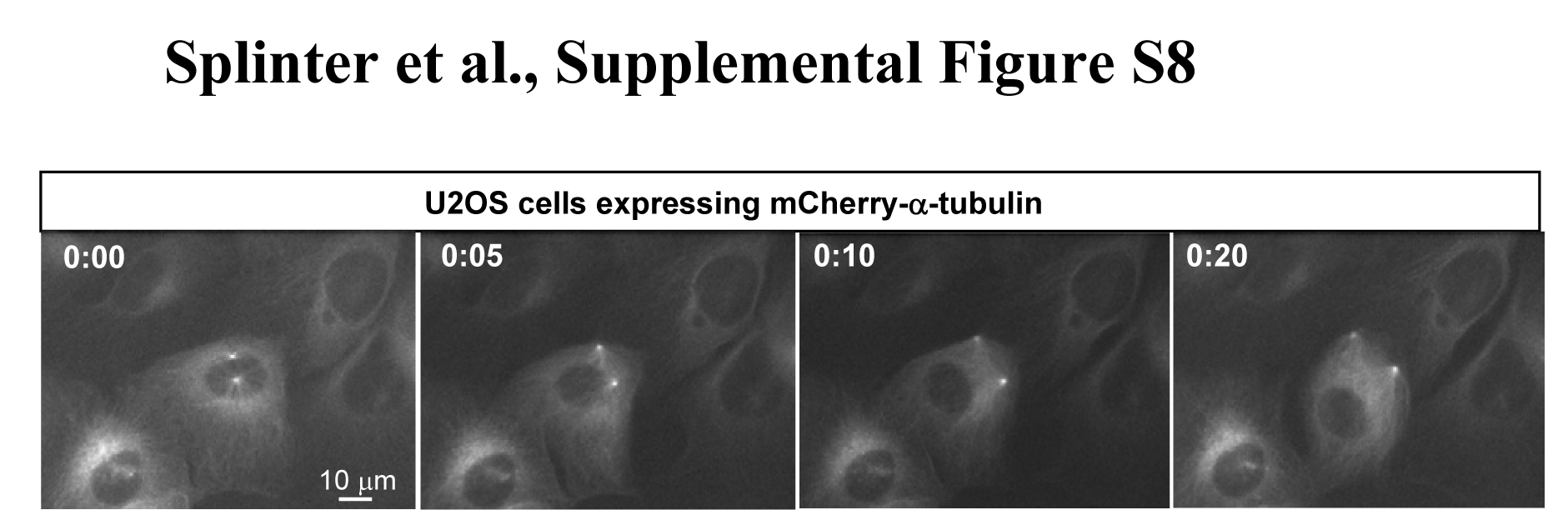

Supplement: Figure S8 — Microinjection of the recombinant coiled coil fragment 1 (CC1) of p150Glued causes rapid separation of centrosomes and the nucleus in prophase cells. U2OS cells stably expressing mCherry-α-tubulin were microinjected with recombinant CC1 at the needle concentration 0.85 mg/ml. Late G2 cells were chosen based on the presence of separated centrosomes. Time t = 0:00 indicates the time-point just prior to injection. (0.29 MB TIF) [file pbio.1000350.s008.tif]

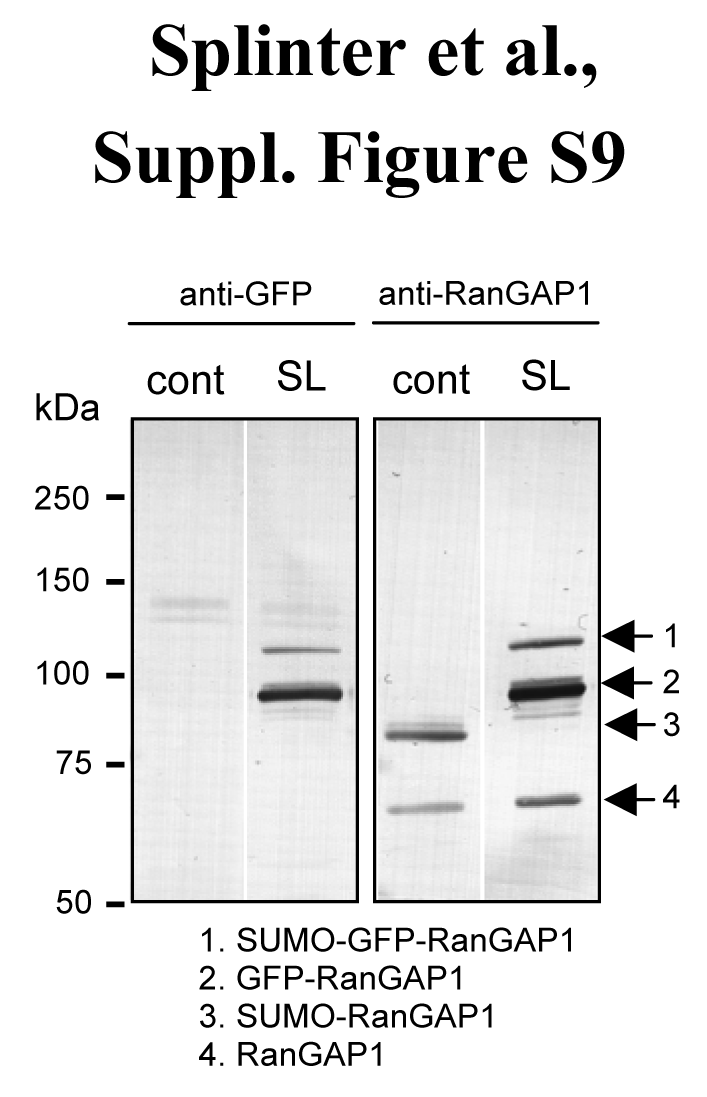

Supplement: Figure S9 — Characterization of a HeLa cell line stably expressing GFP-RanGAP1. Western blots prepared with equal amounts of extracts of control HeLa cells or the stable GFP-RanGAP1 HeLa cell line and incubated with antibodies against GFP or RanGAP1. Note that the expression levels of the fusion protein exceeded the endogenous RanGAP1 levels by approximately a factor of 4; however, the amount of SUMOylated RanGAP1, which is likely to be RanBP2- and NPC-bound [23],[24], was not significantly altered compared to control cells; in the stable cell line, this pool was predominantly represented by the GFP-tagged RanGAP1. (0.14 MB TIF) [file pbio.1000350.s009.tif]

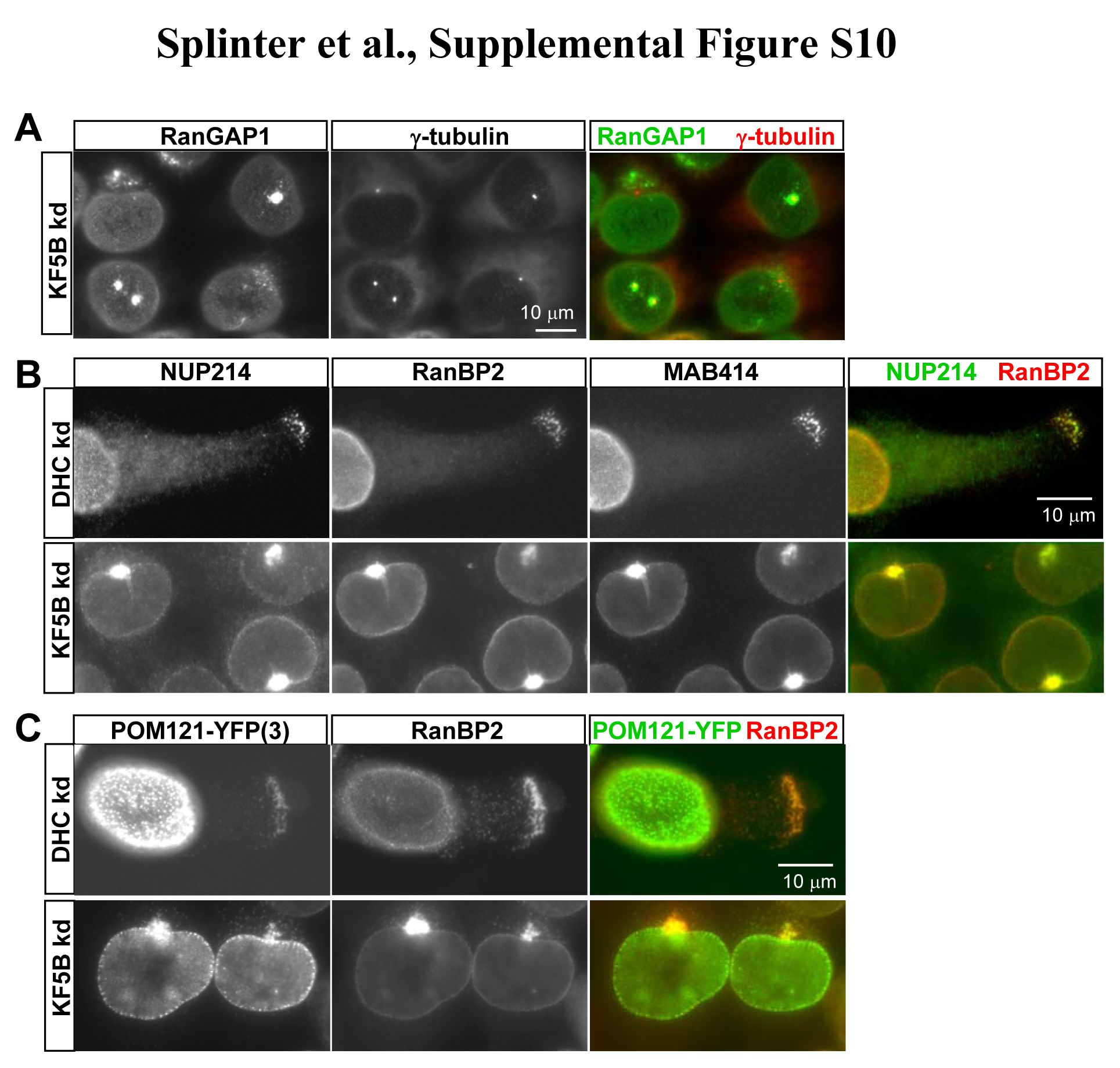

Supplement: Figure S10 — The effect of MT motor depletion on AL distribution. (A) HeLa cells were transfected with siRNAs against KIF5B, fixed with cold methanol 3 d later, and stained for RanBP2 and γ-tubulin. Note that AL accumulate as a single pericentrosomal dot in a cell with a single centrosome and around both centrosomes after their separation. (B) HeLa cells were transfected with KIF5B or DHC siRNAs, fixed with methanol, and stained for endogenous NUP214, RanBP2, and with the MAB414 antibody. (C) HeLa cells stably expressing POM121-YFP(3) were transfected with KIF5B or DHC siRNAs, fixed with paraformaldehyde, and stained with antibodies against RanBP2. Note that all NPC markers show characteristic re-localization to the centrosome or to the cell periphery, supporting the view that these structures are indeed AL. (1.65 MB TIF) [file pbio.1000350.s010.tif]

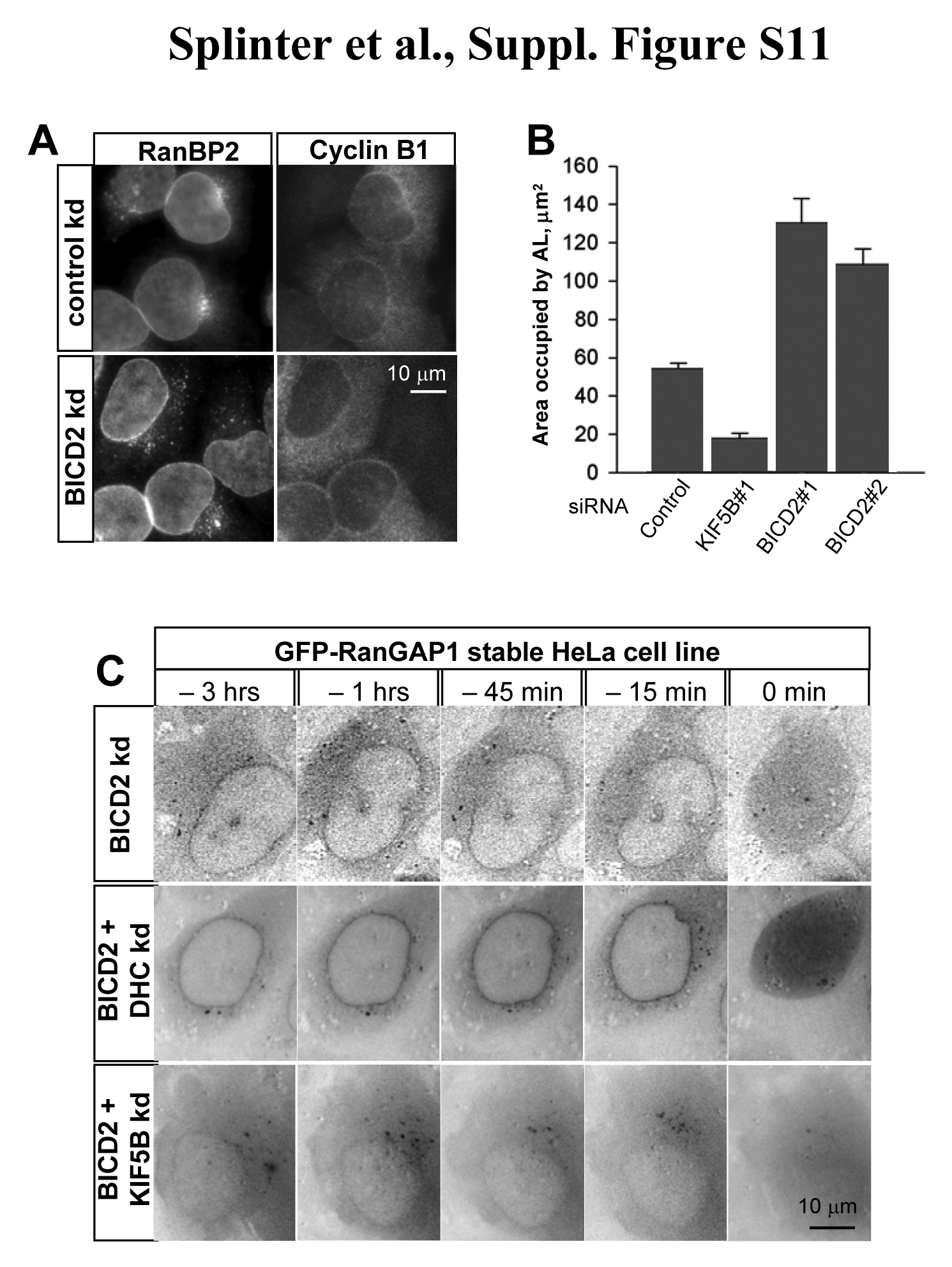

Supplement: Figure S11 — Behavior of AL in BICD2-depleted cells. (A) HeLa cells were transfected with the indicated siRNAs, fixed 3 d later with cold methanol followed by paraformaldehyde, and stained for RanBP2 and cyclin B1. Note that AL are dispersed in BICD2-depleted cyclin B1-positive cells. (B) Total area occupied by AL was measured in ∼25 cyclin B1-positive cells stained as described for (A). Error bars represent SEM. The area is decreased in KIF5B-depleted G2 cells because AL are strongly concentrated in the pericentrosomal region and enlarged in BICD2-depleted cells because AL are dispersed. (C) GFP-RanGAP1 stable HeLa cell line was imaged with a 3 min time interval 2 d after transfection with the BICD2#1 siRNA alone or in combination with siRNAs against DHC#1 or KIF5B#1. 0 min indicates the first frame after NEB. Contrast is inverted. Note that AL remain dispersed and accumulated neither at the cell periphery nor the cell center, even when dynein or KIF5B were co-depleted. (0.93 MB TIF) [file pbio.1000350.s011.tif]

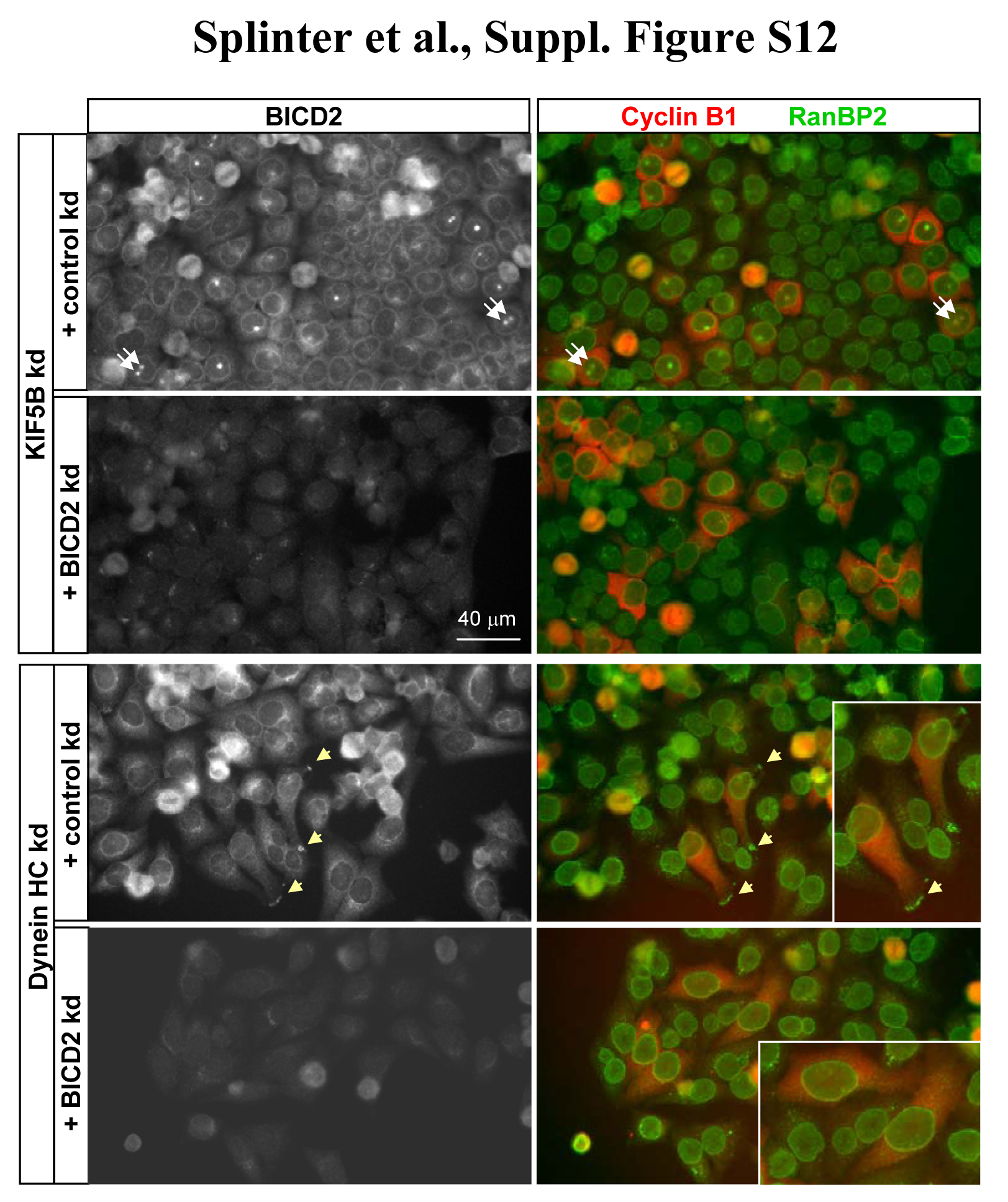

Supplement: Figure S12 — BICD2 depletion blocks re-localization of AL caused by the knockdown of dynein or kinesin-1. HeLa cells were transfected with the KIF5B#1 or DHC#1 siRNAs in combination with the control or BICD2#1 siRNAs, fixed with paraformaldehyde 3 d later, and stained for BICD2, RanBP2 (green in overlay), and cyclin B1 (red in overlay). Insets show enlargement of cyclin B1-positive dynein-depleted cells. Accumulations of AL at the two separated centrosomes in kinesin-1-depleted cells and at the cell periphery in dynein-depleted cells are indicated by arrows. Note that cells showing strong AL displacement to the centrosome or the cell periphery cannot be found in BICD2-codepleted cells. (2.64 MB TIF) [file pbio.1000350.s012.tif]

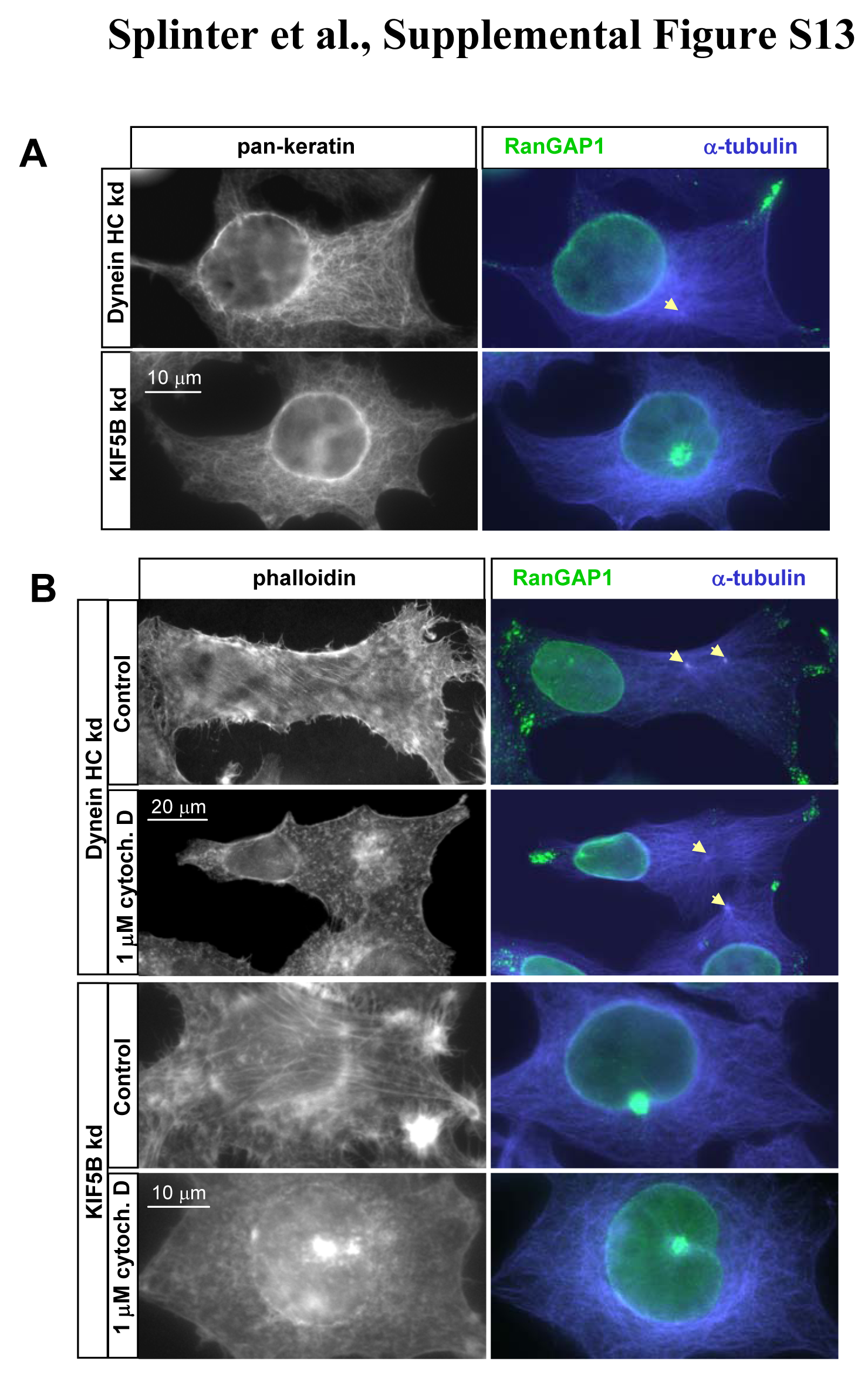

Supplement: Figure S13 — Keratin and actin are not directly involved in G2-specific displacement of nuclei and AL after dynein and kinesin-1 depletion. (A) HeLa cells were transfected with the indicated siRNAs, fixed with cold methanol 3 d later, and stained for keratin, α-tubulin, and RanGAP1. Note that keratin network looks similar in dynein HC and KIF5B-delpeted G2 cells (which are identified by the characteristic position of the AL). This is in agreement with published data indicating that keratin distribution is not significantly affected by microtubule motors [67]. (B) HeLa cells were transfected with the indicated siRNAs; 2 d later, cells were incubated overnight with 1 µm cytochalasin D or left untreated. At 3 d post-transfection, cells were fixed with paraformaldehyde and stained with antibodies to α-tubulin and RanGAP1, and with fluorescent phalloidin, to visualize the actin network. Note that cytochalasin-induced disassembly of actin fibers does not prevent separation of centrosomes (arrows) and nuclei in a dynein-depleted cell or AL aggregation in KIF5B-depleted cell. (1.92 MB TIF) [file pbio.1000350.s013.tif]

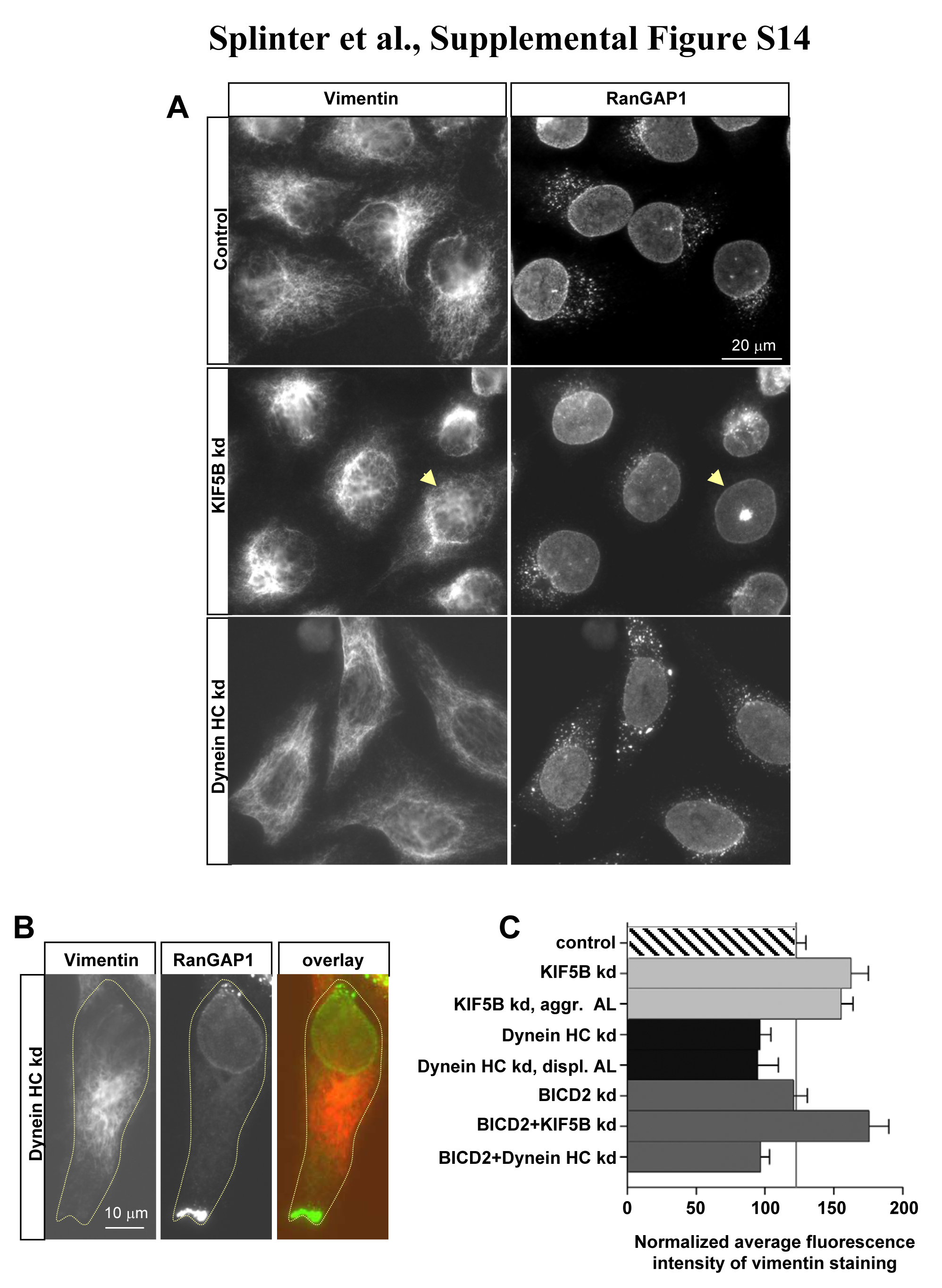

Supplement: Figure S14 — Vimentin distribution is affected by knockdown of dynein and kinesin-1 but does not correlate with G2-specific displacement of nuclei and AL. (A,B) HeLa cells were transfected with the indicated siRNAs, fixed with cold methanol 3 d later, and stained for vimentin and RanGAP1. A cell with the characteristic G2-specific aggregation of AL caused by KIF5B depletion is indicated by an arrow. (B) shows a cell with the characteristic G2-specific peripheral displacement of AL and the nucleus caused by dynein HC depletion; note that vimentin network is not re-localized to the cell corner together with the nucleus. (C) Average fluorescence intensity of vimentin staining in the perinuclear region normalized to the average fluorescence intensity of the whole cell (expressed in %). Intensity of a circular area (3 µm in diameter) was measured in ∼20 cells; background was subtracted. In case of dynein HC and KIF5B knockdowns, two cell populations were analyzed: cells with randomly dispersed AL (corresponding to G1 and S phase) and cells with strongly aggregated (KIF5B depletion) or peripherally located (dynein HC depletion) AL, which are in G2 phase. For the other conditions, cell cycle stages were not discriminated. In agreement with published data, the distribution of vimentin became more concentrated in the center of the cell after KIF5B knockdown and shifted to the cell periphery after dynein knockdown (A,C) [67]. However, in contrast to the positioning of the nuclei and AL, the distribution of vimentin was cell-cycle independent (C). Moreover, in dynein-depleted cells that showed a strong displacement of the nuclei into one cell corner, vimentin network remained in the central part of the cell, indicating that vimentin redistribution is not the underlying cause of the nuclear movement (B). (1.73 MB TIF) [file pbio.1000350.s014.tif]
