# Supplementary material for: Bicaudal D2, Dynein, and Kinesin-1 Associate with Nuclear Pore Complexes and Regulate Centrosome and Nuclear Positioning during Mitotic Entry
Source: PLoS Biol. 2010 Apr 6;8(4):e1000350. doi: 10.1371/journal.pbio.1000350 (PMC2850381; doi:10.1371/journal.pbio.1000350)
Supplement: Table S1 — Identification of BICD2-CT binding partners by mass spectrometry in HeLa cell extract. The table shows the proteins identified with a significant Mascot score in the pull-down with streptavidin beads from an extract of HeLa cells co-expressing Bio-GFP-BICD2-CT (BICD2 amino acids 487–820) and biotin ligase BirA. A pull-down from HeLa cells expressing BirA alone was used as a control (only proteins that displayed significantly higher Mascot score in the Bio-GFP-BICD2-CT lane compared to the control lane are listed). The proteins were separated on 3%–8% polyacrylamide gel (Figure S1); proteins smaller than 50 kDa were not analyzed in this experiment. For each identified protein, the list is filtered for duplicates and shows only the hits with the highest score and most identified peptides. (0.03 MB DOC) [file pbio.1000350.s015.doc]

| **Identified Proteins** | NCBI GI Number | **% coverage** | **Unique peptides** | Mascot Score |
| --- | --- | --- | --- | --- |
| Ran binding protein2 | gi|62088546 | 10,4 | 23 | **1489** |
| Bicaudal D homolog 2 | gi|51479166 | 21,1 | 16 | **1254** |
| Kinesin family member 1C | gi|40254834 | 13,1 | 12 | **826** |
| LL5β | gi|27650425 | 14,3 | 13 | **799** |
| Myosin IC | gi|46430642 | 13,7 | 11 | **763** |
| Non-muscle myosin, heavy polypeptide 9 | gi|12667788 | 7,1 | 10 | **737** |
| Ran GTPase activating protein 1 | gi|119580824 | 15,4 | 8 | **720** |
| Kinesin family member 1B α | gi|41393559 | 10,2 | 9 | **579** |
| CLIP-associating protein 2 | gi|57863301 | 6,6 | 7 | **512** |
| EVI-5 homolog | gi|3093476 | 5,6 | 4 | **293** |
| Nucleoporin 93kDa | gi|41281437 | 5,5 | 3 | **179** |
| Centrosomal protein 170kDa | gi|109255230 | 2,2 | 3 | **170** |
